# Long-term investigation of methane and carbon dioxide emissions in two Italian landfills

**DOI:** 10.1016/j.heliyon.2024.e29356

**Published:** 2024-04-16

**Authors:** L. Brilli, P. Toscano, F. Carotenuto, S. Di Lonardo, P. Di Tommasi, V. Magliulo, A. Manco, L. Vitale, A. Zaldei, B. Gioli

**Affiliations:** a National Research Council of Italy, Institute of BioEconomy (CNR-IBE), Firenze, 50145, Italy; b National Research Council of Italy, Research Institute on Terrestrial Ecosystems (CNR-IRET), Sesto Fiorentino, 50019, Florence, Italy; c National Research Council of Italy, Institute for Agricultural and Forest Systems in the Mediterranean (CNR-ISAFOM), Ercolano, 80056, Naples, Italy

**Keywords:** Eddy covariance, GHGs emissions, Landfill management, Biogas recovery, GHGs budget

## Abstract

Landfills play a key role as greenhouse gas (GHGs) emitters, and urgently need assessment and management plans development to swiftly reduce their climate impact. In this context, accurate emission measurements from landfills under different climate and management would reduce the uncertainty in emission accounting. In this study, more than one year of long-term high-frequency data of CO_2_ and CH_4_ fluxes were collected in two Italian landfills (Giugliano and Case Passerini) with contrasting management (gas recovery VS no management) using eddy covariance (EC), with the aim to i) investigate the relation between climate drivers and CO_2_ and CH_4_ fluxes at different time intervals and ii) to assess the overall GHG balances including the biogas extraction and energy recovery components. Results indicated a higher net atmospheric CO_2_ source (5.7 ± 5.3 g m^2^ d^−1^) at Giugliano compared to Case Passerini (2.4 ± 4.9 g m^2^ d^−1^) as well as one order of magnitude higher atmospheric CH_4_ fluxes (6.0 ± 5.7 g m^2^ d^−1^ and 0.7 ± 0.6 g m^2^ d^−1^ respectively). Statistical analysis highlighted that fluxes were mainly driven by thermal variables, followed by water availability, with their relative importance changing according to the time-interval considered. The rate of change in barometric pressure (dP/dt) influenced CH_4_ patterns and magnitude in the classes ranging from −1.25 to +1.25 Pa h^−1^, with reduction when dP/dt > 0 and increase when dP/dt < 0, whilst a clear pattern was not observed when all dP/dt classes were analyzed. When including management, the total atmospheric GHG balance computed for the two landfills of Giugliano and Case Passerini was 174 g m^2^ d^−1^ and 79 g m^2^ d^−1^ respectively, of which 168 g m^2^ d^−1^ and 20 g m^2^ d^−1^ constituted by CH_4_ fluxes.

## Highlights

• CO_2_ fluxes were mainly driven by soil water content (SWC) and solar radiation.
• Thermal variables were the main driver of CH_4_ fluxes.
• Drought and warm conditions may reduce correlation between SWC and CH_4_ fluxes.
• The rate of change in barometric pressure is inversely correlated with CH_4_ fluxes.
• Gas recovery and combustion process was beneficial in terms of GHG impact.

## 1 Introduction

Methane (CH_4_) is among the most important greenhouse gas (GHG) and the second most abundant after carbon dioxide (CO_2_), accounting for about 20% of global CO_2_-equivalent emissions [1]. This gas, mainly produced by the decay of organic material, can be introduced into the atmosphere by either biogenic (i.e., digestion of food by cattle, organic fermentation etc.) or anthropogenic (i.e., fossil methane extraction and distribution in the oil and gas sectors, etc.) sources. Among the major processes causing CH_4_ emissions, waste disposal is one of the most important, thus making landfills an important source of GHG emissions.

Despite the major attention on curbing methane emissions has focused on the oil and gas sector, the waste sector accounted for 20–27% of all human-related methane emissions [2] and approximately 5% of the global greenhouse budget [3,4]. Landfills emissions can be considered biogenic since methane derives from the fermentation of organic substrates contained in the waste, but they are highly related to anthropogenic processes associated to waste production. Although these data are affected by a large source of uncertainty (about 30%, [5]), these estimates indicate landfills as major greenhouse gas emitters, making an urgent need in their assessment and management to swiftly reduce their climatic impact.

In this context, it is necessary to provide accurate measurements to reduce the uncertainty in GHG emission estimates from landfills, which is often limited by lack of knowledge and experimental data [6,7]. Currently, emission data mostly rely on inventorial calculations based on waste data [8] or modelling approaches [9] that do not or only partly consider landfill conditions (i.e., waste material, age, time of activity, etc.), management (methane vented, biogas recovery for energy production, etc.), and meteorological data (i.e., air temperature, radiation, humidity, changes in barometric pressure, etc.) [10,11].

To quantify GHG emissions from landfills at landfill-level, so as including all those variables which control both the magnitude and the dynamics of landfills GHG emissions, direct measurements collected at different time and space resolution are fundamental. Methodologies such as open and closed chamber can provide noticeable advantages by excluding interference from surrounding CO_2_ and CH_4_ sources whilst have limitations due to low spatial and temporal resolution that make challenging the estimation of annual total methane emissions [12]. Differential Absorption Lidar [13] and tracer gas dispersion methods [8] can cover a wider footprint than ﬂux chambers but, since a significant part of biogas vented to the atmosphere can derive from fugitive emissions, the low temporal resolution could affect the detection of these emissions. Recently remote sensing techniques were proposed to assess methane emitters, but they are still limited to so called super-emitters that do not include sources such as medium scale domestic landfills [14].

The above-mentioned limitations could be overcome using eddy covariance (EC). This technique, largely used in atmospheric and environmental science to determine trace gases exchanges over anthropic and natural ecosystems including urban areas [15,16], agricultural systems [[17], [18], [19]], grasslands [20,21], and forests [22,23], can provide continuous and automated measurements over long periods. Also, EC does not affect in depth and surface soil conditions, thus reducing uncertainties in the fluxes measured from landfill. The major shortcomings of EC concerns site morphology and the footprint area of the measure, which in many cases is not able cover the whole landfill since depending by mast height and meteorological parameters. These factors can reduce the accuracy of the measured fluxes and their capacity to be representative of the whole site [12,24]. Despite these limitations, EC can be suitable for providing continuous and automated measurements of GHG fluxes in heterogeneous environments such as landfills [[24], [25], [26], [27], [28]]. To our knowledge, few long-term measurements have been performed to quantify landfill emissions on a continuous and long-term basis (at least 6 months) with the eddy covariance method [29,30], while other previous studies used eddy covariance only for short-term field campaign ranging from a few hours to some weeks [27,[31], [32], [33]].

In this study, more than one year of long-term high-frequency (i.e., half-hour) data of CO_2_ and CH_4_ were collected in two Italian landfills with contrasting management (e.g., venting the biogas to the atmosphere or collecting it for electric energy recovery) using EC with the aim to: i) determine the relation between climate drivers and CO_2_ and CH_4_ fluxes at different time intervals; ii) assess the overall GHG balance based on the adopted management.

## 2 Materials and methods

### 2.1 Study areas

**Fig. 1:**
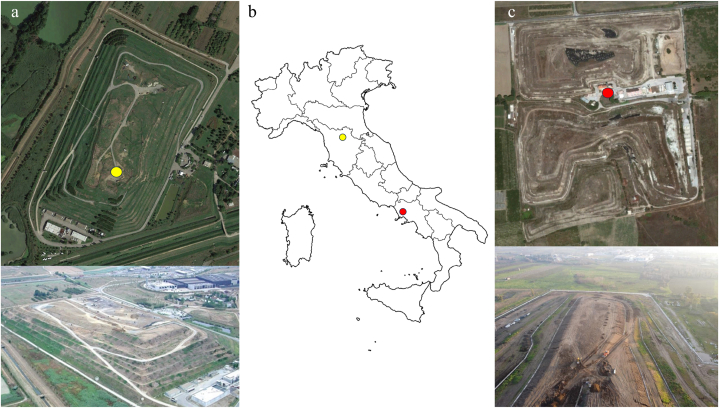
Satellite and aerial images with position of eddy covariance stations (yellow and red stars) at Case Passerini (a) and Giugliano (c), and their localization in Italy (b). (For interpretation of the references to colour in this figure legend, the reader is referred to the Web version of this article.)

The two landfills were located in two distinct geographical areas in Italy. The first site, named Case Passerini (CP), is located in central Italy, within the municipality of Florence (43°48′3″N, 11°10′34″E), while the second is located in southern Italy, within the municipality of Giugliano (Naples, 40°56′48″N, 14°07′02″E) (Fig. 1). The two landfills were similar as far as the type of waste composition, both receiving municipal waste with domestic origin.

CP was active from 1976 to 2009, receiving up to 400 tons per day^−1^ of undifferentiated and heterogeneous commercial and municipal solid waste. The landfill, covering 130,000 m^2^ divided into six lots with a total capacity of 2,100,000 m^3^, is composed by embankments lying and overlapping each other embankments for an average height of about 20 m above the soil level. It is located on a flat plain characterized by clay-sandy soil. The landfill was equipped with a pipeline for leaching water collection and a gas recovery system that, despite the waste reception ended in 2009, continued to extract and use biogas for energy production until 2018. The tanks are equipped with a geomembrane, artificial mineral waterproofing at the bottom and sides, and a bottom drainage system for the leachate. The edaphic soil over the landfill bodies was only about 30 cm depth. Further details of landfill composition can be found in supplementary (Table S1). The vegetation grown up over the site is mainly herbaceous with sporadic shrubs, composed by local native species typical of Mediterranean environment. The landfill is managed by the Municipality of Sesto Fiorentino, Fiorentinambiente, the Quadrifoglio Consortium, and Alia S.p.A.

Giugliano was active from 1980 to 2003 receiving undifferentiated commercial and municipal solid waste, and it covered about 280,000 m^2^. The landfill, located on an alkaline and clay-loam soil, was composed by various bodies named Masseria del Pozzo (north side), Ampliamento Masseria del Pozzo, Ampliamento Schiavi and Novambiente (south side). The average height of the body was about 12 m above the soil level. The landfill of Giugliano had experienced bad management and intensive dumping of toxic waste over a long time, which determined the closure about a decade ago by a court injunction after several years of police investigations. Despite the landfill bodies were covered, nothing is known about the used material neither the type of wastes conferred in this landfill which, coupled with the above-mentioned issues, made this site almost comparable to an abusive dump. The landfill was planned to be equipped with geo-composite capping and collecting tubes for biogas, but the biogas recovery activity was never initiated since the facility was damaged a few years after the landfill opening (theft of poles and other components), so all the produced biogas was vented to the atmosphere. A further description of the site was reported by Ref. [34]. This unfortunate condition offered here the opportunity to investigate the different GHGs balance of the two landfills and accurately quantify the overall emission reductions related to biogas recovery.

### 2.2 Climate data

The long-term climate assessment for the two study areas was performed over the period 1990–2020 using meteorological data obtained from ERA5-land database [35]. Among the several high frequency (1-hr) climatological data included in the dataset, for this study precipitation (mm), mean, maximum, minimum air temperatures (°C), soil temperature (°C) net solar radiation (W m^−2^), air pressure (Pa), dewpoint (°C), soil water content (%), wind direction (°C) and speed (ms^−1^) were extracted. These meteorological data, with a horizontal resolution of roughly 10 km, were used to reduce uncertainties in long-term climate assessment between the two study areas, making comparable the long-term meteorological differences between CP and Giugliano landfills, and to assess their effect on CO_2_ and CH_4_ fluxes when measurements from field sensors were lacking or incomplete, after a site-specific validation with these latter.

### 2.3 Flux tower data and processing

Eddy covariance and site-specific meteorological data were collected over two different years at Case Passerini (10/04/2014–06/05/2015) and Giugliano (18/06/2015–31/10/2016), for a total of 392 and 502 days of measures, respectively. The EC system used for the experiments consisted of a three-dimensional sonic anemometer (Metek, mod. USA-1), a Licor 7500 open path infrared gas analyzer to measure CO_2_ fluxes and a Licor 7700 open path analyzer for CH_4_ measurements. At CP the EC tower was displaced in the center of the landfill over a mast high about 3 m, whilst at Giugliano the EC system was installed over a must high 25 m and located in the middle of the forecourt between the landfill bodies, resulting in a displacement height of 7 m above the height of the bodies (Fig. 1). Ancillary meteorological data, acquired at half-hour frequency and stored within a CR1000 datalogger in both sites, included air temperature (°C), net solar radiation (W m^−2^), relative humidity (%), air pressure (hpa), wind speed (m s^−1^) and direction (°), and rainfall (mm), whilst soil water content (%) and soil temperature (°C) were acquired only at Case Passerini. These latter variables were then used to validate soil water content and soil temperature extracted from ERA5-land database (Fig. S2), which were then adopted for both sites. Furthermore, the rate of change in barometric pressure (d*P*/dt), widely recognized as one of the most important drivers of variability in CH_4_ emissions, was calculated for both sites as the difference between two consecutive hourly air pressure measurements divided by the time interval between measurements (60 min) and correlated with CH_4_ fluxes dynamic.

Footprint calculations were made using the Flux Footprint Prediction (FFP) online data processing tool [36] based on the method developed by Ref. [37]. The footprint calculation required site coordinates, study periods dates, measurement height above ground (m), displacement height (m), roughness length (m), mean wind (ms^−1^), Obukhov length (m), standard deviation of lateral velocity fluctuations after rotation (ms^−1^), friction velocity (ms^−1^), wind direction for rotation of the footprint. Footprint analysis revealed that more than 90% footprint distances are mostly contained within the landfill area (Fig. S1). Raw data collected at high-frequency (20 Hz) were processed using EddyPro Software and then cleaned and harmonized through a post-processing consisting of: i) despiking procedure for detecting and eliminating short-term outliers in the time series and control tests according to Ref. [38]; ii) high pass filtering with linear detrending [39]; iii) corrections of CO_2_ and CH_4_ fluxes for air density fluctuations [40]; iv) gap-filling procedure providing continuous CO_2_ and CH_4_ fluxes records following [41].

Finally, the cleaned and harmonized half-hourly CO_2_ and CH_4_ fluxes were aggregated at hourly, daily, 5-days, 10-days, 15-days, monthly, seasonal, and seasonal diurnal courses (SDC) time steps and then correlated with 10 meteorological variables (mean air temperature (AirT), mean soil temperature (SoilT), precipitation (PP), soil water content (SWC), relative humidity (RH), solar radiation, dewpoint (DewP), air pressure (AirP), wind direction (Wdir) and wind speed (Wspeed)) at the same time-intervals. The relation between fluxes and these meteorological variables was investigated using multiple regression approach and dominance analysis, a procedure that is based on an examination of the R^2^ values for all possible subset models [42].

### 2.4 Remote sensing analysis

The Normalized Difference Vegetation Index (NDVI) was used to explore the vegetation patterns over the two sites during the study periods to evaluate the different vegetation cover and its effect on CO_2_ and CH_4_ fluxes in the two landfills. Time series of NDVI were calculated using the Moderate Resolution Imaging Spectroradiometer (MODIS) at 16-days and 250 m horizontal resolution [43]. MODIS data were accessed through the Google Earth Engine (GEE) Catalog which includes the version 6.1 of the MODIS NDVI dataset. The time series of MODIS NDVI was retrieved between the January 1, 2010 and December 31, 2020, extracting the NDVI value of the grid cell of 250 × 250 m corresponding to the geometric centers of the two landfills. The “SummaryQA” band contained into MODIS data and indicating the overall quality of each pixel was used to mask and remove all data with a quality flag different than 0, with the aim to obtain the most reliable useable data for the analysis.

### 2.5 Biogas recovery and electricity production

The biogas recovered at CP was collected and transported to a power generation unit. Since the CP landfill hadn't any use for the thermal energy eventually produced, thermal energy was not recovered and is not considered here. Biogas flows collected by the gas recovery pipes and transported to the power generation unit and electric energy produced by biogas combustion and transmitted to the grid were reported at monthly intervals (Table S2).

The content of CO_2_ and CH_4_ in the biogas was measured once during the study period, and resulted to be 45% methane, 35% carbon dioxide, 20% other compounds (not measured). These amounts were in line with the typical ranges for landfill biogas, that have a lower CH_4_ content with respect to other types of biogases (e.g., livestock biogas can reach 75% of CH_4_ content). The remaining fraction is likely composed of nitrogen and low amounts of hydrogen and VOC (volatile organic compounds), which were not measured, however. On these bases, in this study, only CO_2_ and CH_4_ were considered for the GHG balance computation, and the biogas composition was assumed to not change during the study period. Yearly amounts of recovered biogas flow and produced electric energy were computed based on the monthly data. Given the non-contemporary study period between the sites (13 and 17 months at CP and Giugliano, respectively), daily data were firstly aggregated at monthly time-step and months with two samples were then averaged to derive a single data per month.

### 2.6 Assessment of GHG balance

The GHG balance of each site was computed including direct emissions to the atmosphere through landfill surface, emissions related to biogas recovery and associated energy production, and avoided emissions due to electric energy production. In this latter process, the methane fraction of the biogas was converted to electric energy by a combustion engine, where methane was transformed to CO_2_ and vented to the atmosphere whilst the CO_2_ fraction of the biogas was directly vented to the atmosphere.

The full GHG balance was computed as:

(1) FC_tot_ = F_CO2_EC_ + F_CH4_EC_ + F_CO2_RB_ + F_CO2_MC_ + F_CO2_ REP_

where FC_tot_ is the total GHG balance in kg-CO_2_eq m^−2^ y^−1^; F_CO2_EC_ is the CO_2_ emitted and measured by eddy covariance; F_CH4_EC_ is the CO_2_ equivalent emission measured by eddy covariance and computed using a CH_4_ global warming potential (GWP) of 28 kg-CO_2_eq m^−2^ y^−1^ for the cumulative impact over 100 years [44]; F_CO2_RB_ is the CO_2_ fraction of the extracted biogas metered at the energy production facility that is vented to the atmosphere; F_CO2_MC_ is the CO_2_ vented to the atmosphere after the methane combustion process (with other trace gases like VOCs considered negligible in terms of GHG balance); F_CO2_REP_ is the avoided emissions due to energy production.

According with the procedure outlined by Ref. [45] for the conversion of generated Italian thermic and electric energy, at Case Passerini the recovered biogas generated electric energy that resulted in avoided GHG emissions based on the energy mix in use in Italy at the time of the measurements, that was equal to 483 gCO_2_eq/KWh. This negative contribution was therefore subtracted from the total GHG balance. Therefore, the final GHG balance was determined in terms of CO_2_eq m^−2^ y^−1^ as follow:

(2) CP_GHG_BAL_ = 1 × CO_2_EC_ + 28 × CH_4_EC_ −483 × CO_2_REP_ + 1 × CO_2_RB_ + 1 × CO_2_MC_

(3) GIUGLIANO _GHG_BAL_ = 1 × CO_2_EC_ + 28 × CH_4_EC_

## 3 Results and discussion

### 3.1 Meteorological conditions

**Fig. 2:**
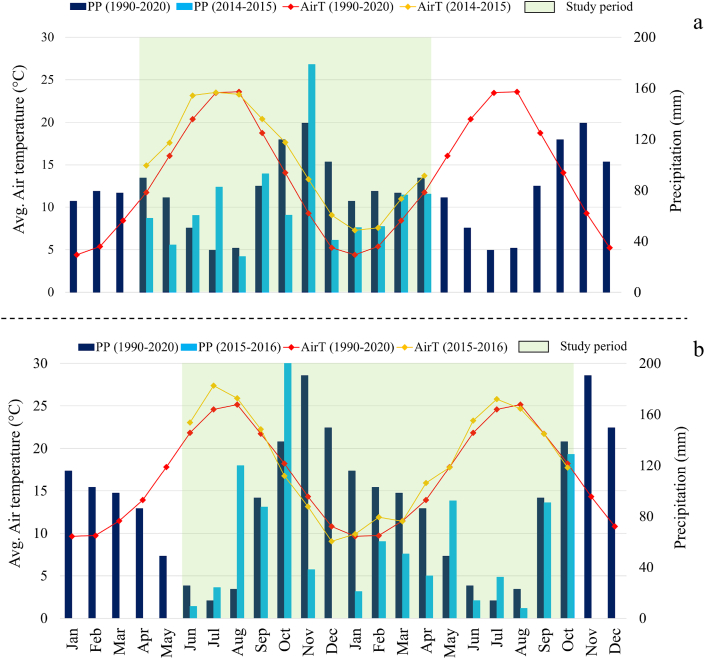
Long term (1990–2020) monthly patterns of average air temperature (red line) and cumulated precipitation (blue histograms) against monthly air temperature (orange line) and precipitation (cyan histograms) during the study periods at (a) Case Passerini (2014–2015) and (b) Giugliano (2015–2016). (For interpretation of the references to colour in this figure legend, the reader is referred to the Web version of this article.)

The pattern of monthly air temperatures observed at CP (Fig. 2a) during the study period (green area) was rather consistent with the long-term climate pattern (1990–2020), with higher temperatures (+3 °C, on average) from September 2014 to April 2015. Monthly cumulated precipitation was found in line with long-term average, with the exception of summer 2014 which was wetter (+44%).

At Giugliano (Fig. 2b) the mean air temperatures during the study period (green area) was consistent with long-term climate (1990–2020) along the whole year, with the only exception of summer 2015 (⁓+1.6 °C, on average). The cumulated monthly precipitation was consistently lower than the long-term average, especially from November 2015 to February 2016 (−438 mm) and in spring 2016 (−58 mm).

Generally, the climate analysis suggested warmer and drier conditions at Giugliano compared to CP, with monthly temperatures about +2 °C higher along the year and lower precipitation in winter and summer.

### 3.2 Daily CO_2_ and CH_4_ fluxes

**Fig. 3:**
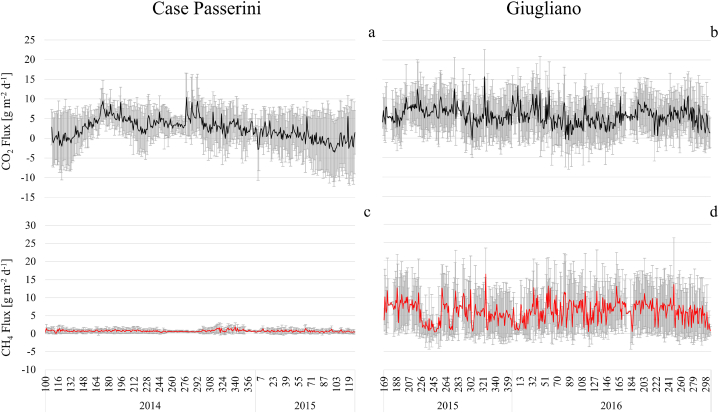
Daily patterns of CO_2_ and CH_4_ fluxes at case Passerini (April 2014 to May 2015) and Giugliano (June 2014 to October 2015) during the study periods. Grey bars represent standard deviation.

The daily patterns of CO_2_ and CH_4_ fluxes at Case Passerini and Giugliano were reported in Fig. 3. At Case Passerini, the CO_2_ fluxes revealed a seasonality, with C-sequestration during springtime in both years and higher emissions in summer and winter. By contrast, at Giugliano the CO_2_ pattern did not show any significant seasonality, resulting into an average higher net CO_2_ source (5.7 ± 5.3 g CO_2_ m^2^ d^−1^) compared to Case Passerini (2.4 ± 4.9 g CO_2_ m^2^ d^−1^). Globally, for the two distinct periods of analysis, the CO_2_ emissions were 952.6 ± 1726.5 and 2870.0 ± 2128.0 g CO_2_ m^2^ at Case Passerini and Giugliano, respectively.

Concerning CH_4_ fluxes, both sites showed mostly stable emissions along the year but considerable differences in magnitude. The CH_4_ fluxes ranged from 0.4 to 16.3 g CH_4_ m^2^ d^−1^ at Giugliano and from 0.07 to 1.9 g CH_4_ m^2^ d^−1^ at Case Passerini, making the landfill of Giugliano an average net CH_4_ source about ten times higher (6.0 ± 5.7 g CH_4_ m^2^ d^−1^) compared to Case Passerini (0.7 ± 0.6 g CH_4_ m^2^ d^−1^). The total CH_4_ emissions during the whole period of investigation were 291.8 ± 185.3 g CH_4_ m^2^ at Case Passerini and 3009.7 ± 2551.7 g CH_4_ m^2^ at Giugliano.

### 3.3 Vegetation recovery

**Fig. 4:**
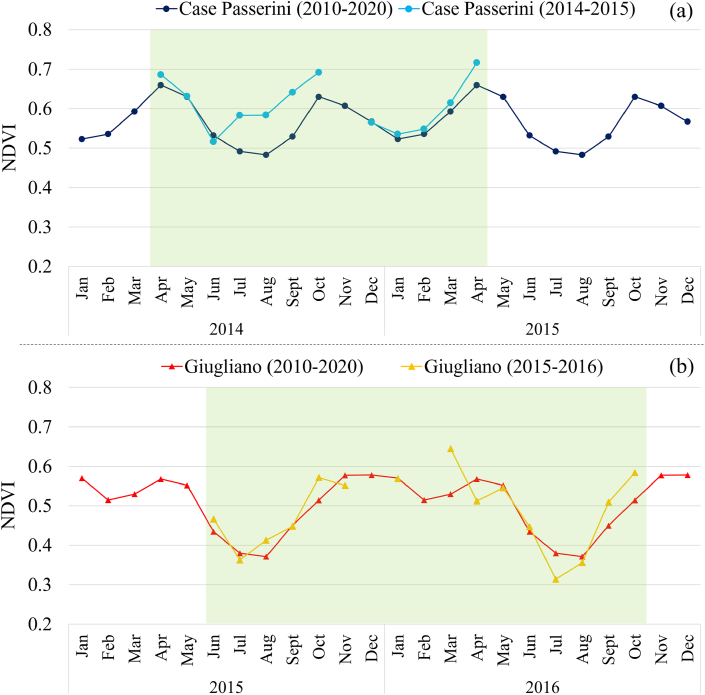
NDVI profile for the period 2010–2020 (NDVI_10yr_) and during the study period at Case Passerini (a) and Giugliano (b).

The NDVI profile of the two landfills for the period 2010–2020 (NDVI_10yr_) showed similar patterns but notable differences in magnitude (Fig. 4). At Case Passerini the NDVI_10yr_ was generally higher (0.56) than that observed at Giugliano (0.50), with the higher peaks observed in April (0.66) and October (0.63), and the minimum in August (0.48); whilst at Giugliano the higher peaks were observed in April (0.57), November and December (0.58), and the minimum in August (0.37).

These differences exacerbated during the study periods, where the NDVI profile of grass vegetation at Case Passerini during 2014–2015 was much higher compared to its NDVI_10yr_ (Fig. 4a). The higher peaks were observed in April (0.69) and October (0.69), and the minimum in June (0.52), with higher values persistently observed from June to October 2014 and during the spring of 2015.

At Giugliano, the NDVI profile of grass vegetation during 2015–2016 was almost in line with its NDVI_10yr_ but markedly lower than that observed at Case Passerini. The higher peaks were observed in October 2015 (0.57), and March (0.64) and October (0.58) 2016, whilst the lowest in July 2015 (0.36) and 2016 (0.31).

Generally, the major NDVI differences between the two sites were observed during the growing season, usually considered for Mediterranean grass vegetation from April to October, where the NDVI was much higher at Case Passerini (0.63) than Giugliano (0.46). These results were also confirmed by the differences between nighttime and daytime hours of CO_2_ fluxes in the two landfills (Fig. S3), where a clear C-sequestration was observed during daytime at Case Passerini compared to the nighttime values, whilst none or only little differences were observed at Giugliano.

### 3.4 Seasonal CO_2_ and CH_4_ fluxes and meteorological variables

**Fig. 5:**
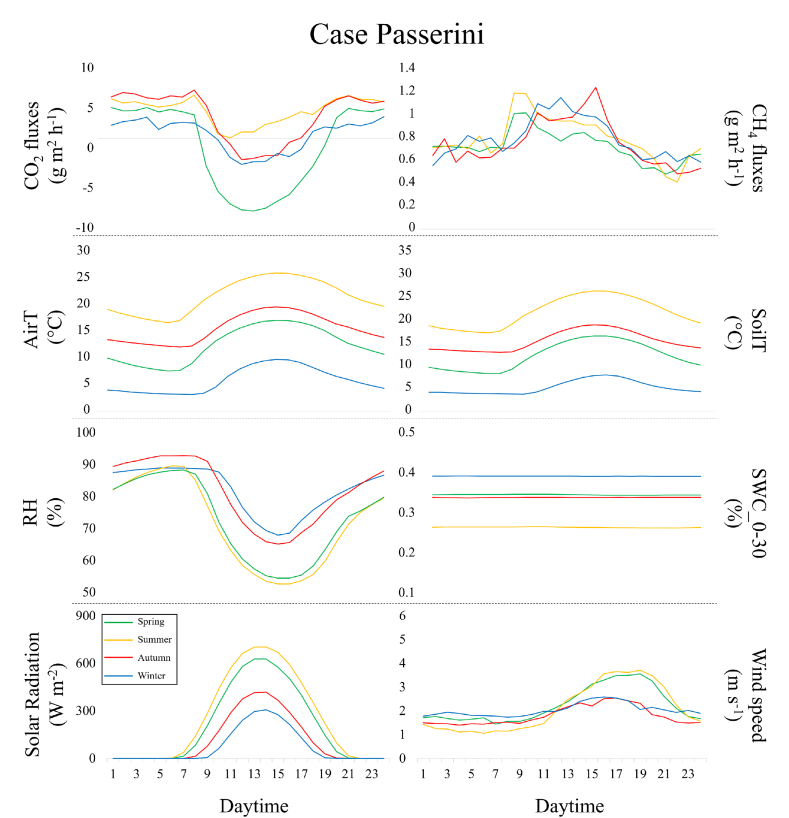
Seasonal diurnal courses (spring, summer, autumn and winter) of C-fluxes (CO_2_ and CH_4_) and measured meteorological variables (mean air (AirT) and soil (SoilT) temperature, relative humidity (RH), soil water content (SWC), solar radiation and wind speed) during the study period at Case Passerini.

**Fig. 6:**
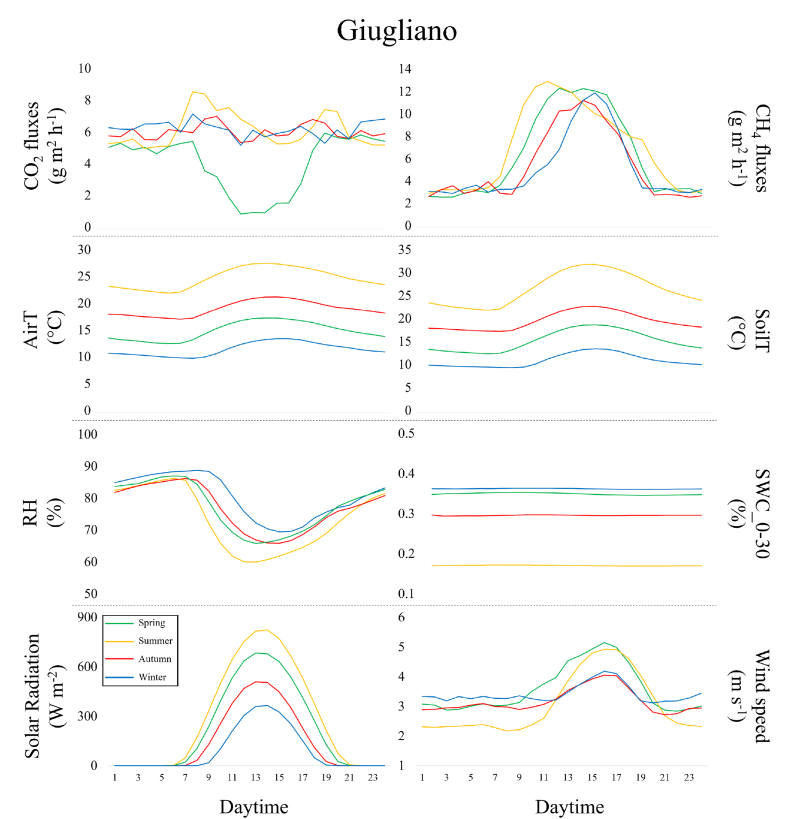
Seasonal diurnal courses (spring, summer, autumn and winter) of C-fluxes (CO_2_ and CH_4_) and measured meteorological variables (mean air (AirT) and soil (SoilT) temperature, relative humidity (RH), soil water content (SWC), solar radiation and wind speed) during the study period at Giugliano.

The CO_2_ flux hourly dynamic at Case Passerini (Fig. 5) reproduced the typical gaussian shape in all seasons, with the larger carbon uptake during the middle of the day in springtime (−7.9 ± 5.8 g m^2^ h), followed by winter (−2.1 ± 4.0 g m^2^ h) and autumn (−1.5 ± 3.7 g m^2^ h) (Table S3). By contrast, the summer seasonal diurnal course (SDC) showed continuous CO_2_ emissions, with the minimum emissions peak in the morning (1.2 ± 5.1 g m^2^ h) (Table S3). On average, the spring SDC was the closest to neutrality (0.1 ± 3.9 g CO_2_ m^2^ d^−1^), whilst it showed the highest emissions (4.7 ± 3.4 g CO_2_ m^2^ d^−1^) in summer. By contrast, none of the CO_2_ hourly dynamic at Giugliano showed C-uptake, resulting in an average continuous CO_2_ emission (Fig. 6), with the lowest CO_2_ emissions peak observed during the middle of the day in springtime (0.8 ± 5.9 g m^2^ h), (Table S3). This pattern was translated into a slightly lower average daily CO_2_ emissions in spring (4.0 ± 5.0 g CO_2_ m^2^ d^−1^) compared to summer (6.1 ± 4.3 g CO_2_ m^2^ d^−1^), autumn (6.0 ± 5.1 g CO_2_ m^2^ d^−1^) and winter (6.2 ± 5.2 g CO_2_ m^2^ d^−1^).

The CH_4_ hourly dynamic at Case Passerini (Fig. 5) reproduced a similar gaussian shape in all seasons, with the highest emissions peak during early morning in summer (1.2 ± 0.7 g m^2^ h) and spring (1.0 ± 0.8 g m^2^ h), during late morning in winter (1.1 ± 0.8 g m^2^ h), and in the afternoon in autumn (1.2 ± 0.8 g m^2^ h) (Table S4). Globally, the average SDC were similar for all seasons (0.7 ± 0.6 to 0.8 ± 0.6 g CH_4_ m^2^ d^−1^). This pattern was clearly observed also at Giugliano but larger in magnitude (Fig. 6), with the highest emission peak during early morning in summer (12.9 ± 7.7 g m^2^ h) and the lowest emission peak in the middle of the day in autumn (11.2 ± 8.0 g m^2^ h) (Table S4). The average SDC emissions were similar for all seasons, with highest in summer (6.9 ± 4.8 g CH_4_ m^2^ d^−1^) and the lowest in winter (5.1 ± 4.8 g CH_4_ m^2^ d^−1^).

The seasonal diurnal courses of air temperatures showed minimum values in early morning and highest in the middle of the day in all seasons, with higher variability (i.e., difference between maximum and minimum air temperature = Δ) among the seasons at Case Passerini (Δ22.9 °C, range 2.8–25.7 °C) than Giugliano (Δ17.8 °C, range 9.7–27.5 °C). Also, spring and autumn diurnal courses of air temperatures were relatively closer at Case Passerini compared to those observed at Giugliano. Solar radiation showed the same pattern between seasons and sites, but lower magnitude at Case Passerini than Giugliano.

Major differences were observed in the seasonal diurnal courses of relative humidity (RH) and soil water content (SWC) between the sites. At Case Passerini the relative humidity showed consistent differences between seasons, with winter and autumn (81.5 and 81.9%, on average) clearly more humid than spring and summer (72.6 and 71.3%, on average), whilst the highest SWC was in winter (0.39%, on average) and the lowest in summer (0.26%, on average), with spring and autumn reporting similar levels (0.34%, on average). By contrast, at Giugliano the RH showed lower variability among the seasons, with highest values in winter (80.4%, on average), lowest in summer (73.5%, on average), and similar condition in spring (77.3%, on average) and autumn (76.9%, on average). The SWC was generally lower than that observed at Case Passerini, with higher values in winter and spring (0.36 and 0.35%, on average), and the lowest in summer (0.17%, on average).

Finally, wind speed showed similar pattern between the sites, with higher speed found in warmer (spring and summer) than colder (autumn and winter) seasons. The maximum wind speed was observed in spring at Case Passerini (3.5 m s^−1^) and in summer at Giugliano (4.9 m s^−1^). Globally, wind speed was higher at Giugliano in all seasons (3.3 m s^−1^, on average) compared to those observed at Case Passerini (2 m s^−1^, on average).

### 3.5 Spatial emission variability and effect of changes in barometric pressure

**Fig. 7:**
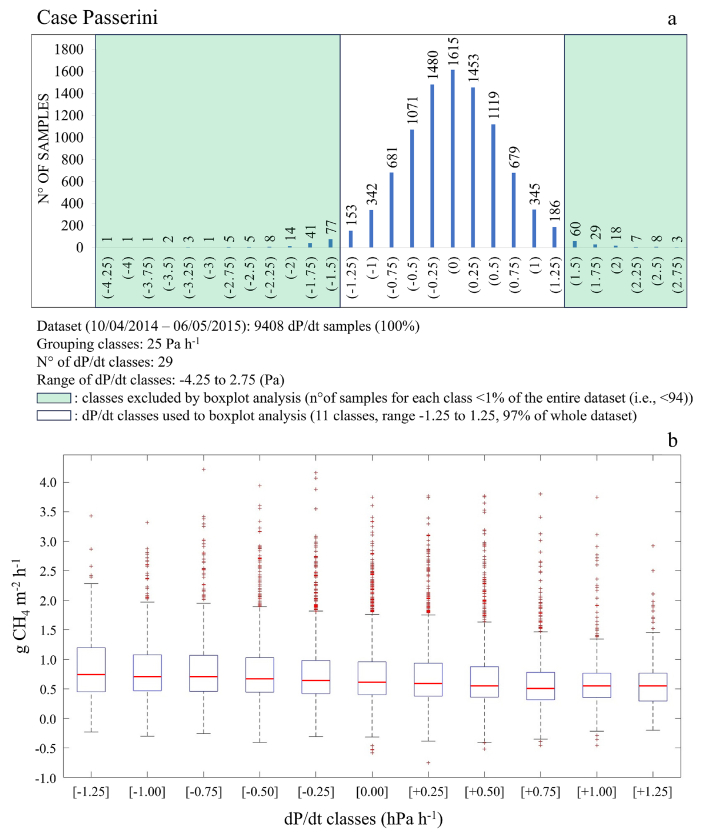
a) Histograms grouping all CH_4_ hourly fluxes recorded at Case Passerini and grouped in in dP/dt classes of 25 Pa, for a total of 29 dP/dt classes ranging from −4.25 to 2.75. The classes contained a number of samples (CH_4_ fluxes) < 1% of the entire dataset (i.e., <94) were reported within a green area and excluded by the boxplot analysis; b) boxplot analysis carried out on the 11 remaining dP/dt classes (−1.25 to 1.25) covering the 97% of the CH_4_ dataset. Error bars represent the variability of the measurements calculated as the standard deviation of the mean. The central line in each box is the median, whilst the bottom and top edges of the box indicate the 25th and 75th percentiles, respectively. The circles represent outliers. (For interpretation of the references to colour in this figure legend, the reader is referred to the Web version of this article.)

The GHGs emission as a function of wind direction (Fig. S4) was accounted to detect possible dependency of both CO_2_ and CH_4_ fluxes to specific source areas. The analysis did not show any significant prevailing hotspot located inside the footprint area of the EC station, with median CO_2_ and CH_4_ fluxes approximately similar among the other area sectors. Concerning the effect of change in barometric pressure, at Case Passerini (Fig. 7) d*P*/dt was found to have a clear effect on CH_4_ emission (R^2^ = 0.97; Fig. S5). Specifically, the highest CH_4_ fluxes were found over periods with decreasing barometric pressure, while lower values were observed when barometric pressure increased. These changes were little, however, with CH_4_ fluxes measured by EC varying from 0.89 to 0.61 g CH_4_ m^−2^ h^−1^. The pattern of changes in CH_4_ fluxes grouped in d*P*/dt classes of 25 Pa (Fig. 7) included almost all dataset, with the exception for those fluxes included in d*P*/dt classes with a number of samples lower than 1% of the entire dataset (i.e., 94 values).

**Fig. 8:**
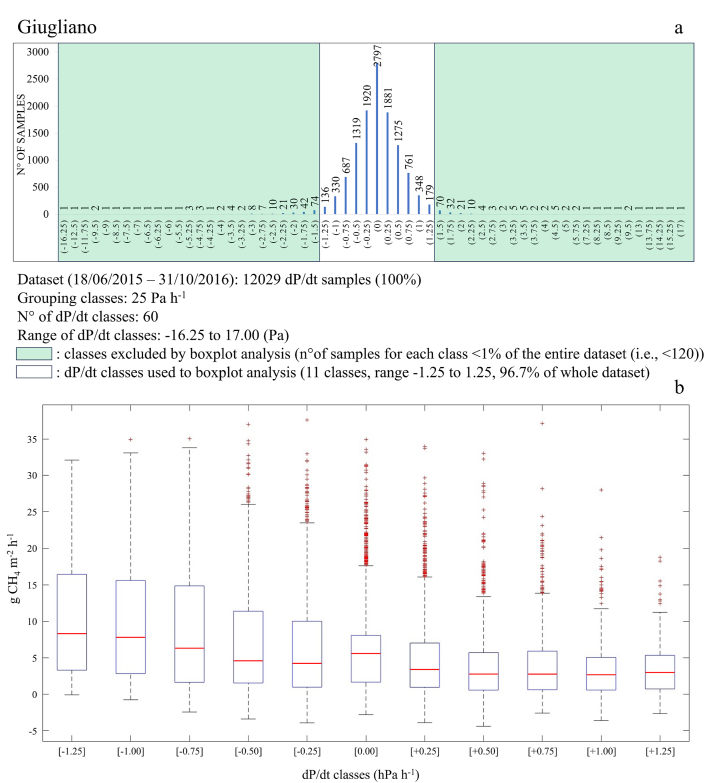
a) Histograms grouping all CH_4_ hourly fluxes recorded at Giugliano and grouped in dP/dt classes of 25 Pa, for a total of 60 dP/dt classes ranging from −16.25 to 17.00. The classes contained a number of samples (CH_4_ fluxes) < 1% of the entire dataset (i.e., <120) were reported within a green area and excluded by the boxplot analysis; b) boxplot analysis carried out on the 11 remaining dP/dt classes (−1.25 to 1.25) covering the 96.7% of the CH_4_ dataset. Error bars represent the variability of the measurements calculated as the standard deviation of the mean. The central line in each box is the median, whilst the bottom and top edges of the box indicate the 25th and 75th percentiles, respectively. The circles represent outliers. (For interpretation of the references to colour in this figure legend, the reader is referred to the Web version of this article.)

A similar relation was observed also at Giugliano (Fig. 8), with d*P*/dt showing a pronounced effect on CH_4_ emission (R^2^ = 0.94; Fig. S5), with highest CH_4_ fluxes found over periods with decreasing barometric pressure and lower values observed when barometric pressure increased. Compared to Case Passerini these changes were greater, with CH_4_ fluxes measured by EC varying from 10.4 to 3.7 g CH_4_ m^−2^ h^−1^. As for Case Passerini, the pattern of changes in CH_4_ fluxes grouped in bins of 25 Pa included almost all dataset with the exception for those fluxes included in classes with a number of samples lower than 1% of the entire dataset (i.e., 120 values; Fig. 8).

### 3.6 Climate regulation of CO_2_ and CH_4_ fluxes

**Fig. 9:**
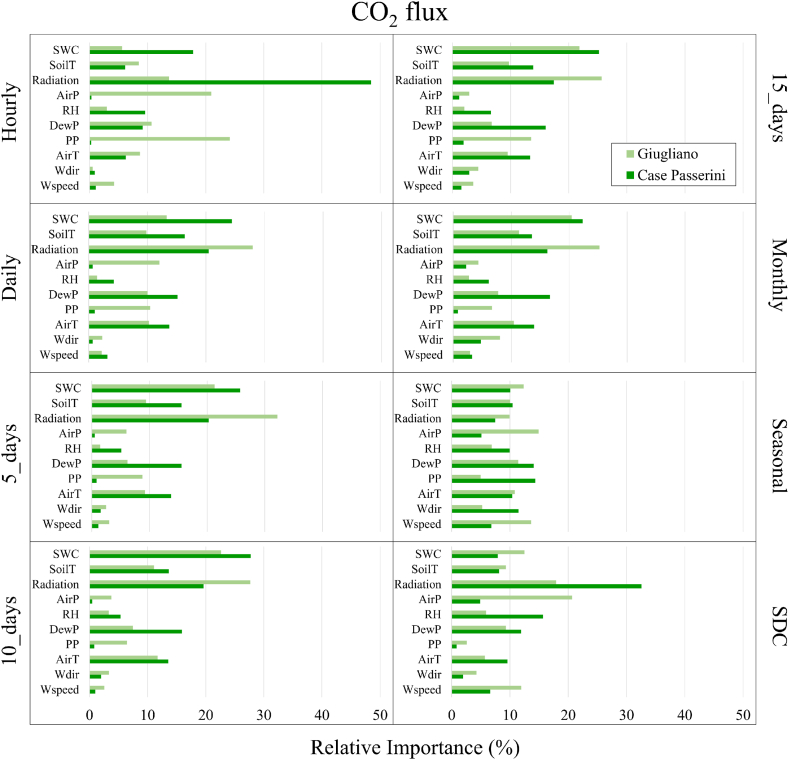
Relative importance (%) of meteorological variables explaining CO_2_ flux at hourly, daily, 5-days, 10-days, 15-days, monthly, seasonal, and seasonal diurnal courses (SDC) time steps.

The relation between CO_2_ and CH_4_ fluxes and all meteorological variables collected within the sites was investigated for the two study areas for the different time-intervals investigated, reporting the relative importance (RI) of each meteorological variable as percentage contribution to the explanation of the total variance (%). Also, the RI of all meteorological variables to CO_2_ and CH_4_ fluxes were accounted at high (hourly and SDC; Fig. S1) and low (daily to season; Fig. S2) frequency and averaged for all selected time-step (Fig. 9), with the rank of meteorological variables empirically defined grouping their RI at 10% step. This rank (R) was established to better define the role of meteorological drivers able to explain the contribution of at least 10% of the CO_2_ and CH_4_ fluxes according to the time resolution of data collection.

For CO_2_ flux, solar radiation, and soil water content (SWC) resulted to be the most important driving factors in the two sites under almost all the time step, following by air and soil temperature, and dewpoint (Fig. 9). Specifically, RI of solar radiation ranged from 7.5% (seasonal) to 48.4% (hourly) at Case Passerini (green bars), and from 9.9% (seasonal) to 32.2% (5-days) at Giugliano (light green bars), whilst the RI of SWC ranged from 7.9% (SDC) to 27.7 (10-days) at Case Passerini, and from 5.6% (hourly) to 22.6% (10-days) at Giugliano. Wind speed and direction, precipitation and air pressure showed a general lower relative importance in both sites (RI<20%), with the only exceptions for precipitation (24.1%) and air pressure (20.9%) at hourly time step, and air pressure at SDC (20.7%), at Giugliano. The RI of the meteorological variables was clearly smoothed at seasonal time step, where the RI of each variable was <20% (Fig. 9).

**Fig. 10:**
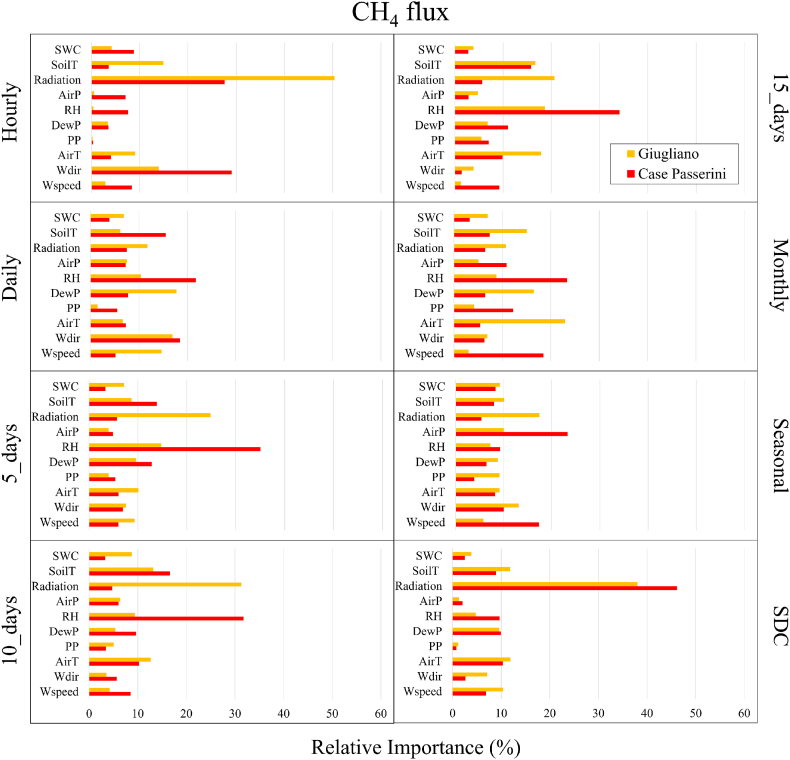
Relative importance (%) of meteorological variables explaining CH_4_ flux at hourly, daily, 5-days, 10-days, 15-days, monthly, seasonal, and seasonal diurnal courses (SDC) time steps.

For CH_4_ flux, the RI of meteorological variables was more complex than that observed for CO_2_ flux, strongly changing according to sites and time-step (Fig. 10). At Case Passerini (red bars) the major meteorological driver was relative humidity (RH), with RI ranging from 7.6% (hourly) to 35.1% (5-days), and lower than 20% under hourly, seasonal (9.2%) and SDC (9.7%). The other meteorological variables with RI>20% were wind direction (29%) and solar radiation (27.6%) at hourly time-step, air pressure (23.3%) at seasonal scale, and solar radiation at SDC (46.2%.). At Giugliano (orange bars), the major meteorological driver clearly was solar radiation, with RH that ranged from 10.7% (monthly) to 50.4% (hourly) and found lower than 20% only under daily (11.8%), monthly and seasonal (17.4%) time-step. All remaining meteorological variables showed RI<20% under all time-steps, with the only exception for air temperature (23%) at monthly time step.

**Fig. 11:**
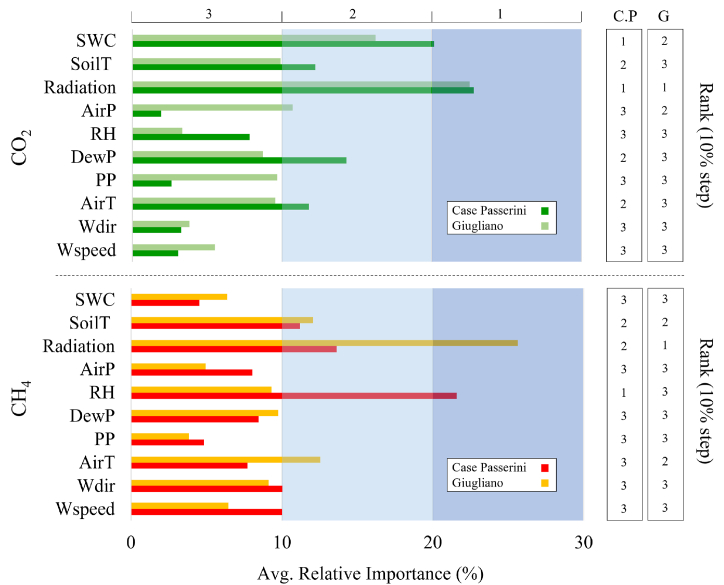
Relative importance (%) of meteorological variables explaining CO_2_ and CH_4_ fluxes as average of all selected time-step. The rank of meteorological variables (1, 2 and 3) was empirically defined grouping at 10% step (white, cyan, and light blue area) their relative importance. (For interpretation of the references to colour in this figure legend, the reader is referred to the Web version of this article.)

Finally, the RI of all meteorological variables to CO_2_ and CH_4_ fluxes were accounted at high (hourly and SDC; Fig. S6) and low (daily to season; Fig. S7) frequency and averaged of all selected time-step (Fig. 11), with the rank of meteorological variables (1, 2 and 3) empirically defined grouping their RI at 10% step (white, cyan and light blue area). Based on the average of all selected time-step, for CO_2_ flux at Case Passerini the highest rank (1) was found for solar radiation (22.8%) and SWC (20.1%), followed by dew point (14.3%), soil (12.2%) and air temperature (11.8%), whilst at Giugliano the highest rank was found only for solar radiation (22.5%), followed by SWC (16.2%) and air pressure (10.7%).

For CH_4_ flux, at Case Passerini the highest rank (1) was found for RH (21.6%), followed by solar radiation (13.6%) and soil temperature (11.2%), whilst at Giugliano the highest rank was found for solar radiation (25.6%), followed by air (12.5%) and soil temperature (12.1%).

Globally, solar radiation showed the highest rank under 3 of 4 sites, resulting the main driver of CO_2_ fluxes together with SWC, and the main driver of CH_4_ fluxes together with soil and air temperature, and relative humidity.

### 3.7 GHG balance

The total biogas recovered in CP was 3,179,764 Normal Cubic Meter per year (Nm^−3^y^−1^), whilst the electric energy transmitted to the grid was 4,307,229 Kwh_e_ y^−1^, resulting in a conversion efficiency of biogas into electric energy of 1.35 Kwh_e_/Nm^−3^. The CO_2_ and CH_4_ standard density were computed at 1.87 and 0.66 kg/Nm^−3^, respectively. Given the respective volumetric content in the biogas mixture, actual CO_2_ and CH_4_ density in the biogas was computed at 0.654 kg CO_2_/Nm^−3^ and 0.297 kg CH_4_/Nm^−3^. Since methane was burned into the IC engine to produce electricity, it was emitted to the atmosphere as 0.841 kg CO_2_/Nm^−3^. Doing so, the total CO_2_ emitted from biogas to the atmosphere was 1495 kg CO_2_/Nm^−3^ of biogas, composed of 0.654 kg CO_2_/Nm^−3^ originally present in the biogas, and 0.841 kg CO_2_/Nm^−3^ derived from the combustion of methane present in the biogas. By multiplying for the total biogas yearly flow, a total of 4,753,747 kg CO_2_ y^−1^ was emitted to the atmosphere, composed of 2,079,566 kg CO_2_ y^−1^ of native CO_2_ and 2,674,181 kg CO_2_ y^−1^ of CO_2_ derived from CH_4_ combustion, and no methane was emitted to the atmosphere from the CP recovered biogas.

The electricity production induced a negative GHG term expressed in CO_2_ equivalent related to emissions that were avoided to produce the same amount of energy. By multiplying the total energy yearly production (i.e., 4,307,229 Kwhe y^−1^) for an emission factor of 484 g CO_2_eq/kWh_e_ (specific for the energy mix of Italy), a total of 2,084,698 kg CO_2_eq y^−1^ negative emission was obtained for the CP landfill. The total GHG emission related to the recovered biogas fraction was therefore equal to 2,669,049 kg CO_2_eq y^−1^. The GHG balance for each landfill was finally calculated as g CO_2_ eq m^−2^ y^−1^ to fit with the emissions measured by eddy covariance tower, which were 887 (CO_2_ flux) and 7535 (CH_4_ flux) g CO_2_ eq m^−2^ y^−1^ at Case Passerini, and 2086 (CO_2_ flux) and 61,272 (CH_4_ flux) g CO_2_ eq m^−2^ y^−1^ at Giugliano.

**Fig. 12:**
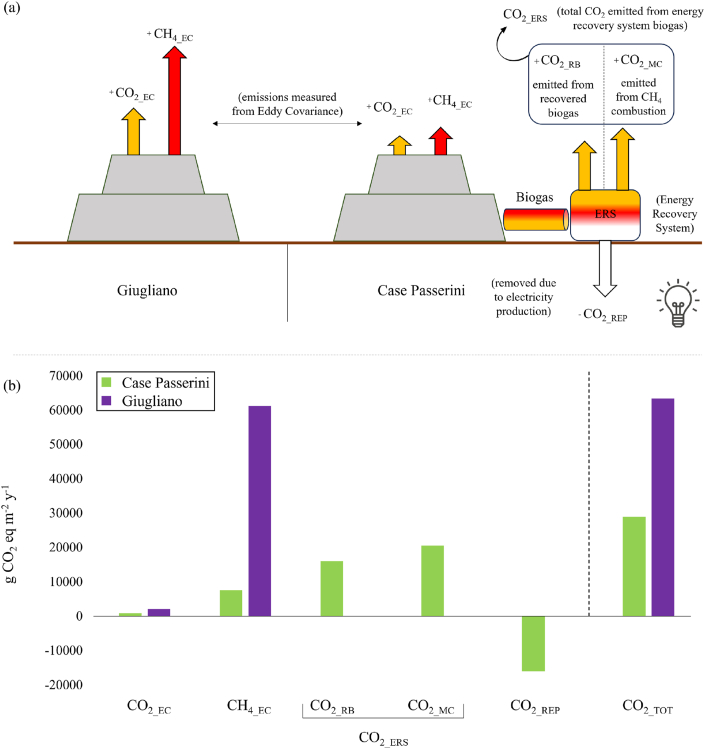
Graphical representation of (a) the GHG balance at Giugliano (purple) and Case Passerini (green) and (b) related emissions (g CO_2_eq. m^−2^ y^−1^) for the different components in the two sites. (For interpretation of the references to colour in this figure legend, the reader is referred to the Web version of this article.)

Globally, the GHG balance was higher at Giugliano (174 g m^2^ d^−1^ or 63,359 g CO_2_eq. m^−2^ y^−1^) than CP (79 g m^2^ d^−1^ or 28,953 g CO_2_eq. m^−2^ y^−1^), with the electricity production that induced a negative GHG emissions of 16,056 g CO_2_eq. m^−2^ y^−1^ at CP (Fig. 12).

## 4 Discussion

### 4.1 Climate regulation of fluxes

The CO_2_ and CH_4_ fluxes generated in landfills provide a substantial contribution to global GHG emissions [46]. Currently, few studies addressed high-frequency long-term dynamics of these fluxes, whilst the understanding of their main driving factors and the quantification of these emissions is essential to develop new methodologies, approaches, and strategies to improve the climate sustainability of landfills. In this work, the seasonal diurnal pattern of CO_2_ fluxes reproduced a typical gaussian shape in all seasons and sites, with lowest C-emissions in springtime and the highest in summer. These patterns clearly reflected the behavior of the vegetation present on the soil surface of landfills, which increased the C-uptake in springtime, when water and radiative forcing were optimal for plant growth, whilst reduced its efficiency under seasons characterized by limiting growing factors such as drought conditions (i.e., summer) or low air temperatures and solar radiation (i.e., winter). The RI analysis between meteorological variables and CO_2_ fluxes confirmed the primary role of SWC and solar radiation as the most important driving variables of CO_2_ fluxes (Fig. 11).

Differences in magnitude and patterns of seasonal courses of CO_2_ fluxes between the two sites were likely related to the different climate conditions and landfill management of the study sites. Specifically, the prolonged drought conditions in summertime observed at Giugliano may have increased ecosystem respiration of the grass cover compared to that of CP, leading lower C-sequestration capacity [47]. This was confirmed by daytime versus nighttime CO_2_ fluxes analysis (Fig. S3), where Giugliano landfill did not show considerable differences between daytime and nighttime patterns as instead observed at Case Passerini, and by the NDVI analysis (Fig. 4), where the profile of grass vegetation during the growing season for the two study periods was much lower at Giugliano (0.46) than Case Passerini (0.63).

Concerning CH_4_ fluxes, changes in barometric pressure influenced CH_4_ patterns and magnitude, with CH_4_ emissions reduced when d*P*/dt > 0 and increased when d*P*/dt < 0. These findings were in line with literature, where a similar dependence on CH_4_ emissions to d*P*/dt was shown [25,[48], [49], [50], [51]]. As suggested by Ref. [24], this effect may be due to an advective transport mechanism caused by the dP/dt influence on the difference between internal waste pressure and that atmospheric. In this study, this effect was clearly found for dP/dt classes ranging from −1.25 to +1.25 hPa h^−1^, whilst looking at the whole dataset a clear pattern was not observed (Figs. S8 and S9). Since air pressure was often constant along the year and characterized by only few abrupt rises or drops in wintertime in both locations (Fig. S10), the extreme dP/dt classes contained few CH_4_ values, making the dP/dt effect on CH_4_ fluxes clearly observable only in a narrow range of dP/dt classes (−1.25 to +1.25 hPa h^−1^), where the higher number of samples provided a more robust information about the CH_4_ flux pattern.

The seasonal diurnal courses of CH_4_ reproduced a gaussian shape in all seasons in both sites, with the highest emissions peak which moved from early morning in summer and spring, to the afternoon in winter and autumn. This dynamic suggested that the combination of thermal variables and water availability were the main drivers for the methane formation processes [52,53]. Specifically, whilst in summertime the effect of solar radiation on soil CH_4_ processes was maximum in the first hours of the morning, when water content was available due to nighttime dew deposition, in autumn and wintertime the solar radiation effect was maximum in the afternoon, when it was able to warm soil and water availability was not a limiting factor. The effect of solar radiation and, generally of all thermal variables (air and soil temperature) to CH_4_ fluxes, was particularly intense likely due to the low soil thickness in both landfills, which allowed to enhance the temperature in the whole soil layer. This effect likely increased the decomposition rate of organic matter and the metabolic rate of microbial communities, which increases as temperature increase in terms of soil respiration per amount of microbial biomass C, thus leading an increase in CH_4_ production [54,55].

Among the thermal variables, despite the effect of temperature was expected to be inversely correlated with CH_4_ emissions due to their effect on microbial CH_4_ oxidation activity [50,56,57], this condition was not observed neither at CP nor Giugliano (Fig. S11). By contrast, a weak positive correlation between CH_4_ fluxes and temperature was found. This is in line with [58], which also reported a well not explainable weak positive correlation, thus suggesting an indirect effect on soil CH_4_ processes. The effect of SWC on CH_4_ fluxes was found to be little in both sites, playing a slight major role at Giugliano than CP. The relatively weak correlation between SWC and CH_4_ flux is unexpected, since differences in water content of the soil along the season are expected to influence gas permeability/diffusivity, and in turn microbial oxidation of CH_4_ to CO_2_ in the soil. A plausible explanation could be that warm and drought reduced the correlation between CH_4_ fluxes and environmental factors, weakening the sensitivity of CH_4_ fluxes to SWC [59]. Also, the CH_4_ production may occurs at deeper layers within the landfill substrates, therefore resulting not completely coupled with the upper soil hydrology estimated by SWC from ERA5-land dataset.

### 4.2 Management effect on GHG balance

The GHG balance provided by the landfills of Giugliano and CP was 63,359 and 28,953 g CO_2_eq. m^−2^ y^−1^, of which 61,272 and 7535 g CO_2_eq. m^−2^ y^−1^ constituted by CH_4_ fluxes. These data were comparable with other studies on landfill emissions carried out with different methodologies. Using eddy covariance method [31], at Oak Ridge landfill (USA) reported a total GHG emissions of 61,520 g CO_2_eq. m^−2^ y^−1^, whilst at Ammassuo municipal landfill in Finland [29], reported a total GHG emission of 473,986 g CO_2_eq. m^−2^ y^−1^. Again, in USA at the Bluff Road Landfill [30] measured 223,274 g CO_2_eq. m^−2^ y^−1^ of emissions, whilst [24] in the abandoned Skellingsted landfill (Denmark) reported a total emission that ranged from 1261 to 151,372 g CO_2_eq. m^−2^ y^−1^. Using the Atmospheric Tracer Method (ATM) [60], in seven active Swedish landfills with gas recovery systems obtained CH_4_ fluxes measures ranging from 33,762 to 187,975 g CO_2_eq. m^−2^ y^−1^, whilst [8] reported CH_4_ fluxes ranging from 6387 to 120,450 g CO_2_eq. m^−2^ y^−1^ over 15 Danish landfills with gas extraction systems [61]. using enclosure methods over different uncontrolled landfills in the surrounding area of Moscow found the highest CH_4_ emission rate of 292,000 g CO_2_eq. m^−2^ y^−1^ [62]. comparing ATM with enclosure techniques in two landfills in the USA, found CH_4_ emissions ranging from 83,037 to 1,186,250 g CO_2_eq. m^−2^ y^−1^ for an active site with and without biogas recovery, respectively. [63], using the accumulation chamber method over a 5-year period of investigation at the Municipal Solid Waste landfill of Legoli (Italy) estimated an average GHG emission of 437,857 g CO_2_eq. m^−2^ y^−1^ of which 375,000 from CH_4_ fluxes and 62,857 g CO_2_eq. m^−2^ y^−1^ provided by CO_2_ fluxes [64]. retrieved single CH_4_ emissions from four closely located landfills in Italy, including Giugliano, by means of airborne measurements, finding a mean values for each landfill ranging from 159,687 to 365,912 g CO_2_eq. m^−2^ y^−1^.

In this study, the GHG balance of the two landfills was able to assess how much the gas recovery and combustion process was beneficial in terms of GHG impact. Considering a scenario in which the CP biogas would have been directly vented to the atmosphere without methane combustion, as for Giugliano, the total GHG emissions at CP would have been much higher compared to that currently observed. Specifically, the total emission would have been 8.08 kg CO_2_eq/Nm^−3^ of biogas, composed of 0.65 kg CO_2_/Nm^−3^ of direct CO_2_ emission, and 7.42 kg CO_2_ eq/Nm^−3^ of direct methane emission. In this case, by multiplying for the total biogas yearly flow of 3,179,764 Nm^−3^y^−1^, a total of 25,689,313 kg CO_2_ eq y^−1^ would be emitted to the atmosphere from venting the biogas and resulting in 206,033 g CO_2_eq. m^−2^ y^−1^. These higher GHG emissions estimated at CP under unmanaged scenario compared to the GHG emissions measured at Giugliano may be likely explained by the different activity time span of the two landfills. Since the CP landfill was active until 2009, the waste likely contained more organic and biodegradable components and less recalcitrant materials such as lignin or other compounds compared to the landfill of Giugliano, which closed in 2003, resulting in a faster and higher rate of decomposition and, in turn, C-emissions. Therefore, adopting gas recovery and electricity production the GHG impact was 28,953 g CO_2_eq. m^−2^ y^−1^ instead of 206,033 g CO_2_eq. m^−2^ y^−1^ that would have been emitted without the recovery, resulting in a reduction of about 86%.

To further improve the GHG balance of a managed landfill like CP and approaching the carbon neutrality, cogeneration (e.g., Combined Heat and Power, CHP) could be an effective option, provided that a use for the thermal energy is present. In the case study of CP, the hypothetical thermal production estimated by multiplying the electric energy transmitted to the grid (4,307,229 Kwhe y^−1^) for a factor of 1.2 (https://www.biogasworld.com/product/biogas-management/ges-jenbacher-gas-engines/), would result in a thermal production of 5,168,675 Kwht that, when multiplied for the emission factor of −0.28 kg CO_2_eq per kWh (average for heat produced in EU 15; [65]) would turn into an additional negative GHG term of 1,447,229 kg CO_2_eq y^−1^. This would result into a total GHG balance of 17,821 g CO_2_eq. m^−2^ y^−1^ instead of 206,033 g CO_2_eq. m^−2^ y^−1^, resulting in a further GHG reduction of about 91.4%.

### 4.3 Insights and future challenges

The relation between CO_2_ and CH_4_ fluxes and specific meteorological variables is found to be weaker when time intervals are aggregated. This pattern, reducing the weight of each single variable compared to the total, make hard to detect the impact of each single meteorological variables on fluxes dynamics. However, the understanding of the effect of meteorological drivers on fluxes is essential especially in the perspective of current climate mitigation and energy policies, since expected changes in climate conditions may increase emissions from landfills, therefore opening to develop new policies which may take in account the potential increased production of energy from biogas or specific site-management options to reduce landfill surface emissions (i.e., soil cover, earthworks timing, etc.). Long-term high frequency data of CO_2_ and CH_4_ fluxes and meteorological variables could be also useful to improve the parametrization of current landfill emission models or to develop new models at high temporal resolution. Modelling approach is indeed essential to determine in advance changes in patterns and magnitude of landfill emissions according to site localization, type of residue and management, playing a key role in the perspective of the development of specific climate mitigation options and energy policies over different areas worldwide. These tools, however, did not often consider a sufficiently high time and space resolution data of GHG fluxes and landfill boundary conditions, also excluding soil or climate drivers for gaseous transport or seasonal methanotrophic activity in different soils [10], resulting in under- or overestimation of GHG fluxes compared to measurements. For example [11], comparing modeled emissions using four emissions reporting protocols (i.e., IPCC, EPA GHGRP, CARB and SWICS) over three calendar years from a young landfill with no gas collection system observed a consistently overestimation of annual methane emissions by a factor ranging from 4 to 31 [8]. showed that, for 15 Danish landfills, the methane emissions reported to the European Pollutant Release and Transfer Register (E-PRTR) or the Danish EPA were, on average, more than 5 times greater than the measured emissions [66]. in four U.S. landfills reported that model predicted methane emissions were from 2 to 7 times greater than the measured. Therefore high-frequency data, helping to better accounting variations in the emission rates during time, can contribute to improve emission processes within models and to reduce the uncertainty in their estimates, making these tools more reliable for the planning of waste management strategies such as composting and biomethane production.

Finally, since biogas is produced by the biological degradation of different types of substrates such as biomass, primarily agricultural substrates such as manure, cover and energy crops, and waste from towns and villages, which are fermented by bacteria producing biogas in a multi-stage process, it is debated if biogas should be considered carbon neutral once CO_2_ only is emitted to the atmosphere. As a matter of fact, the emitted CO_2_ comes from biogenic sources, therefore it was previously sequestered from the atmosphere by plants photosynthesis. The approach we adopted here is a pure atmospheric budget of GHG leaving the landfill and emitted to the atmosphere, while different approaches involving the assessment of indirect emissions such as those related to waste transport and disposal or inorganic waste disposal not generating a direct emission in the landfill, can be adopted within a LCA framework [67].

Overall, results from this study highlighted the importance of high-frequency measurements of landfill emissions coupled with field measurements and management information to monitor dumping sites. This approach should be applied over all dumping sites to discriminate site-specific patterns of GHG emissions, but also to better define the relative associated health risks and energy opportunities for local communities where dumping sites are present.

## 5 Conclusions

In the perspective to assess GHG emissions from landfills, accurate approaches for measuring CO_2_ and CH_4_ fluxes would provide reliable information on sustainable management of these systems reducing uncertainties in their estimates. In this study, the application of eddy covariance techniques over two landfills with contrasting management overcame those limitations observed short-term field campaigns, where measurements are often scarce and under representative of the emissions dynamics of the site. Specifically, EC approach provided high-frequency data of CO_2_ and CH_4_ fluxes that were used to obtain a real estimation of the total GHG balance of the two landfills as well as to correlate these fluxes with meteorological drivers to discern effect at specific-selected time-intervals. Statistical analysis suggested that the timing and magnitude of CO_2_ and CH_4_ fluxes were mainly driven by change in barometric pressure, and by thermal variables (solar radiation and temperature) and water availability, depending on the time-interval of assessment. The CO_2_ fluxes showed a clear seasonality at Case Passerini, where the presence of grass cover along the year partly limited CO_2_ emissions due to higher C-fixation in the growing period (i.e., spring), resulting in a lower CO_2_ emission source (2.4 ± 4.9 g CO_2_ m^2^ d^−1^) compared to Giugliano (5.7 ± 5.3 g CO_2_ m^2^ d^−1^), whilst the CH_4_ fluxes showed a seasonal response in both sites, with the biogas recovery system strongly that influenced pattern and magnitude of the CH_4_ emission at Case Passerini, which resulted about ten times lower (0.7 ± 0.6 g CH_4_ m^2^ d^−1^) than Giugliano (6.0 ± 5.7 g CH_4_ m^2^ d^−1^). Globally, the GHG balance provided by the landfills of Giugliano and CP were 63,359 and 28,953 g CO_2_eq. m^−2^ y^−1^, of which 61,272 and 7535 g CO_2_eq. m^−2^ y^−1^ provided by CH_4_ fluxes, while considering a scenario in which the CP biogas would have been directly vented to the atmosphere without methane combustion, the total GHG emissions at CP would have been 206,033 g CO_2_eq. m^−2^ y^−1^, resulting more than three-times higher than Giugliano. This suggested as the presence of an adequate biogas recovery and thermal conversion facility is fundamental to strongly decrease the total emission and should be mandatory in developing actual and forthcoming waste management strategies, that may also include alternative uses of biogenic waste such as composting or biomethane production.

## Data availability statement

Authors declare that data associated with this study are not deposited into a publicly available repository and that will be made available only request.

## Complete ethical statement

All applicable ethical requirements are carefully observed and followed.

## CRediT authorship contribution statement

**L. Brilli:** Writing – original draft, Methodology, Formal analysis, Data curation, Conceptualization. **P. Toscano:** Writing – original draft, Methodology, Conceptualization. **F. Carotenuto:** Software, Formal analysis, Data curation. **S. Di Lonardo:** Methodology, Investigation, Data curation. **P. Di Tommasi:** Writing – review & editing. **V. Magliulo:** Writing – review & editing. **A. Manco:** Writing – review & editing. **L. Vitale:** Writing – review & editing. **A. Zaldei:** Software, Methodology, Data curation. **B. Gioli:** Writing – original draft, Supervision, Investigation, Conceptualization.

## Declaration of competing interest

The authors declare that they have no known competing financial interests or personal relationships that could have appeared to influence the work reported in this paper.
