# Supplementary material for: Long-term investigation of methane and carbon dioxide emissions in two Italian landfills
Source: Heliyon. 2024 Apr 16;10(8):e29356. doi: 10.1016/j.heliyon.2024.e29356 (PMC11033122; doi:10.1016/j.heliyon.2024.e29356)
Supplement: Multimedia component 1 [file mmc1.docx]

Supplementary material of

“Impacts of meteorological drivers and management on CO_2_ and CH_4_ budgets in two Italian landfills”.

| Phases ID | Description |
| --- | --- |
| 1 | Reinforcing geogrille (110 kN/m2, 70*70 mm); |
| 2 | Non-woven fabric layer of 300 gr/m2; |
| 3 | High quality silica sand layer with thickness of 50 cm; |
| 4 | Compacted clay layer of 50 cm; |
| 5 | High-density polyethylene (HDPE) layer with thickness of 1,5 mm; |
| 6 | Geocomposite draining layer for water infiltration drainage and capitation |
| 7 | Superficial layer (1 m) of which 0.3 edaphic soil |
| 8 | Inter-row grassing and shrub (sparse) plantation |

Tab. S1. Details of landfill construction at Case Passerini

| **Year** | **Month** | **Biogas (m^3^ y^-1^)** | **Energy (KWhe)** |
| --- | --- | --- | --- |
| 2014 | Apr. | 289050 | 413400 |
|  | May | 293135 | 403800 |
|  | June | 281025 | 376100 |
|  | July | 291825 | 385400 |
|  | Aug. | 297215 | 406600 |
|  | Sept. | 264765 | 348300 |
|  | Oct. | 279965 | 373900 |
|  | Nov. | 279390 | 382300 |
|  | Dec. | 266935 | 364200 |
| 2015 | Jan. | 276864 | 374900 |
|  | Feb. | 232021 | 302400 |
|  | Mar. | 227910 | 319300 |
|  | Apr. | 217820 | 292700 |
|  | *May* | *211805* | 281800 |
| Total |  | *3709725* | *5025100* |
| Total/yr |  | *3179764* | *4307229* |

Tab. S2. Biogas flows collected by the gas recovery pipes and transported to the power generation unit and electric energy produced by biogas combustion and transmitted to the grid.

| **Daily courses CO_2_ (g m^2^)** | | | | | | | | |
| --- | --- | --- | --- | --- | --- | --- | --- | --- |
| **Hour** | **Case Passerini** | | | | **Giugliano** | | | |
| **- - -** | **Spring** | **Summer** | **Autumn** | **Winter** | **Spring** | **Summer** | **Autumn** | **Winter** |
| 0 | 5.0±3.5 | 6.1±2.2 | 6.4±3.6 | 2.8±4.2 | 5.0±5.0 | 5.3±3.6 | 5.8±4.9 | 6.3±5.3 |
| 1 | 4.6±2.8 | 5.6±2.5 | 6.9±3.2 | 3.3±3.6 | 5.3±4.4 | 5.3±3.7 | 5.7±5.2 | 6.2±4.9 |
| 2 | 4.6±3.4 | 5.8±3.1 | 6.7±3.0 | 3.5±3.8 | 4.9±5.8 | 5.6±3.9 | 6.2±4.3 | 6.2±4.7 |
| 3 | 5.0±2.8 | 5.4±2.9 | 6.2±4.0 | 3.8±3.3 | 5.0±4.8 | 5.0±4.5 | 5.5±5.0 | 6.5±4.5 |
| 4 | 4.5±3.5 | 5.1±2.8 | 6.1±3.8 | 2.3±3.2 | 4.6±4.4 | 5.1±4.8 | 5.5±4.8 | 6.5±5.8 |
| 5 | 4.8±3.7 | 5.3±2.8 | 6.5±3.1 | 3.0±3.2 | 5.1±4.1 | 5.1±4.7 | 6.1±4.6 | 6.6±5.2 |
| 6 | 4.5±3.4 | 5.7±2.9 | 6.3±3.5 | 3.2±2.8 | 5.3±4.2 | 6.5±4.3 | 6.1±5.0 | 6.0±5.2 |
| 7 | 4.1±3.8 | 6.6±2.9 | 7.2±3.8 | 3.1±3.7 | 5.4±4.6 | 8.5±4.9 | 6±5.5 | 7.1±4.8 |
| 8 | -2.3±4.4 | 4.6±3.9 | 5.3±3.2 | 2.2±4.2 | 3.5±5.1 | 8.4±5.2 | 6.8±4.7 | 6.5±5.2 |
| 9 | -5.5±4.8 | 1.7±4.1 | 1.9±3.7 | 1.0±3.2 | 3.2±6.1 | 7.3±4.9 | 7.0±5.5 | 6.3±4.6 |
| 10 | -7.0±4.3 | 1.2±5.1 | 0.5±4.0 | -1.2±3.9 | 1.9±6.8 | 7.5±5.3 | 6.2±6.0 | 6.1±4.8 |
| 11 | -7.8±5.0 | 2.0±4.6 | -1.5±3.7 | -2.1±4.0 | 0.8±5.9 | 6.8±5.0 | 5.3±5.8 | 5.2±5.1 |
| 12 | -7.9±5.8 | 2.0±4.5 | -1.3±3.9 | -1.7±4.8 | 0.9±6.3 | 6.4±4.4 | 5.4±5.7 | 6.1±6.5 |
| 13 | -7.6±5.0 | 2.9±4.5 | -1.0±3.5 | -1.7±5.2 | 0.9±6.1 | 5.8±4.6 | 6.1±5.3 | 5.7±5.9 |
| 14 | -6.7±5.3 | 3.3±4.5 | -1.0±3.8 | -0.7±4.5 | 1.5±6.6 | 5.3±4.4 | 5.8±4.6 | 5.9±5.8 |
| 15 | -5.9±4.8 | 3.8±4.7 | 0.7±2.4 | -1.1±3.4 | 1.5±5.4 | 5.3±4.2 | 5.8±4.9 | 6.1±5.1 |
| 16 | -4.1±4.7 | 4.5±4.0 | 1.2±2.5 | -0.2±3.2 | 2.7±5.0 | 5.6±4.3 | 6.5±4.9 | 6.4±4.8 |
| 17 | -2.3±4.4 | 4.2±3.6 | 2.9±2.9 | 2.0±2.8 | 4.8±4.2 | 6.3±3.9 | 6.8±5.2 | 5.9±5.0 |
| 18 | 0.3±3.1 | 5.3±3.7 | 5.2±3.4 | 2.6±3.3 | 5.9±4.0 | 7.4±4.1 | 6.6±4.7 | 5.3±4.8 |
| 19 | 3.8±2.4 | 6.2±2.6 | 6.0±3.8 | 2.5±3.3 | 5.6±3.9 | 7.3±3.6 | 5.7±5.1 | 6.1±5.1 |
| 20 | 4.9±2.5 | 6.5±1.8 | 6.5±3.9 | 3.0±4.0 | 5.5±4.5 | 5.7±3.5 | 5.6±5.0 | 5.6±5.1 |
| 21 | 4.6±3.0 | 6.1±2.1 | 6.0±3.9 | 2.7±3.7 | 5.8±4.5 | 5.4±3.5 | 6.1±4.3 | 6.6±4.9 |
| 22 | 4.5±3.7 | 6.0±2.9 | 5.6±3.7 | 3.1±3.0 | 5.6±4.3 | 5.2±4.0 | 5.8±5.2 | 6.7±5.4 |
| 23 | 4.9±2.9 | 5.7±2.6 | 5.8±3.8 | 3.9±3.6 | 5.4±3.9 | 5.2±3.5 | 5.9±5.1 | 6.8±5.4 |

Tab. S3. Hourly CO_2_ (g m^-2^) fluxes at Case Passerini and Giugliano during spring, summer, autumn and winter.

| **Daily courses CH_4_ (g m^2^)** | | | | | | | | |
| --- | --- | --- | --- | --- | --- | --- | --- | --- |
| **Hour** | **Case Passerini** | | | | **Giugliano** | | | |
| **- - -** | **Spring** | **Summer** | **Autumn** | **Winter** | **Spring** | **Summer** | **Autumn** | **Winter** |
| 0 | 0.7±0.6 | 0.7±0.5 | 0.6±0.5 | 0.5±0.4 | 2.6±3.0 | 2.9±2.9 | 2.6±3.5 | 3.1±3.7 |
| 1 | 0.7±0.5 | 0.7±0.5 | 0.8±0.8 | 0.7±0.6 | 2.6±2.8 | 3.2±3.3 | 3.2±3.7 | 3.0±3.8 |
| 2 | 0.7±0.5 | 0.7±0.5 | 0.6±0.5 | 0.7±0.5 | 2.6±3.4 | 3.2±3.3 | 3.6±4.0 | 2.9±3.6 |
| 3 | 0.7±0.5 | 0.7±0.5 | 0.7±0.5 | 0.8±0.5 | 2.9±3.2 | 3.2±3.0 | 2.9±3.9 | 3.3±3.5 |
| 4 | 0.7±0.6 | 0.8±0.7 | 0.6±0.5 | 0.8±0.6 | 3.1±3.1 | 3.2±3.4 | 3.2±4.1 | 3.6±3.7 |
| 5 | 0.7±0.6 | 0.7±0.5 | 0.6±0.5 | 0.8±0.6 | 3.0±4.3 | 3.4±3.0 | 3.9±5.1 | 3.0±3.4 |
| 6 | 0.7±0.6 | 0.7±0.5 | 0.7±0.7 | 0.7±0.6 | 3.6±3.3 | 4.4±4.6 | 2.9±3.8 | 3.2±3.6 |
| 7 | 1.0±0.8 | 1.2±0.6 | 0.7±0.5 | 0.7±0.6 | 5.1±5.3 | 7.7±6.6 | 2.8±3.3 | 3.3±3.2 |
| 8 | 1.0±0.7 | 1.2±0.7 | 0.8±0.7 | 0.8±0.6 | 7.0±6.6 | 10.8±8.0 | 4.4±4.5 | 3.6±3.9 |
| 9 | 0.9±0.7 | 1.0±0.5 | 1.0±0.7 | 1.1±0.8 | 9.5±7.2 | 12.4±8.3 | 6.5±6.4 | 4.7±6.3 |
| 10 | 0.8±0.6 | 0.9±0.5 | 0.9±0.6 | 1.0±0.7 | 11.3±7.6 | 12.9±7.7 | 8.3±7.4 | 5.5±5.8 |
| 11 | 0.8±0.6 | 0.9±0.5 | 1.0±0.7 | 1.1±0.8 | 12.3±7.9 | 12.4±7.0 | 10.3±8.9 | 6.8±6.2 |
| 12 | 0.8±0.5 | 0.9±0.5 | 1.0±0.5 | 1.0±0.7 | 11.9±6.7 | 11.9±6.3 | 10.4±8.3 | 9.4±7.0 |
| 13 | 0.8±0.5 | 0.9±0.5 | 1.1±0.6 | 1.0±0.7 | 12.3±7.1 | 10.9±5.8 | 11.2±8.0 | 11.2±7.6 |
| 14 | 0.8±0.5 | 0.9±0.5 | 1.2±0.8 | 1.0±0.6 | 12.0±7.5 | 10.0±5.7 | 10.8±7.4 | 11.9±7.9 |
| 15 | 0.8±0.4 | 0.8±0.4 | 0.9±0.5 | 0.9±0.6 | 11.7±7.8 | 9.5±5.1 | 9.3±6.7 | 10.9±7.9 |
| 16 | 0.7±0.4 | 0.8±0.4 | 0.7±0.4 | 0.7±0.5 | 9.7±7.2 | 8.7±4.7 | 8.1±6.5 | 8.5±6.0 |
| 17 | 0.6±0.5 | 0.7±0.5 | 0.7±0.4 | 0.7±0.5 | 7.9±5.9 | 8.0±4.1 | 6.1±5.3 | 5.6±5.2 |
| 18 | 0.5±0.4 | 0.7±0.5 | 0.6±0.4 | 0.6±0.5 | 5.2±4.2 | 7.7±4.7 | 4.1±4.6 | 3.4±3.9 |
| 19 | 0.5±0.5 | 0.6±0.4 | 0.6±0.4 | 0.6±0.5 | 3.1±3.1 | 5.6±4.0 | 2.8±3.4 | 3.3±3.6 |
| 20 | 0.5±0.4 | 0.4±0.4 | 0.6±0.4 | 0.7±0.5 | 3.3±3.3 | 4.2±3.8 | 2.8±3.4 | 3.3±3.6 |
| 21 | 0.5±0.6 | 0.4±0.4 | 0.5±0.3 | 0.6±0.3 | 3.3±3.6 | 3.1±3.1 | 2.7±3.6 | 3.0±3.9 |
| 22 | 0.6±0.6 | 0.6±0.5 | 0.5±0.3 | 0.6±0.6 | 3.3±3.3 | 3.0±3.0 | 2.6±3.5 | 3.0±3.9 |
| 23 | 0.6±0.6 | 0.7±0.5 | 0.5±0.4 | 0.6±0.5 | 2.9±3.6 | 3.1±3.0 | 2.7±4.0 | 3.2±3.7 |

Tab. S4. Hourly CH_4_ (g m^-2^) fluxes at Case Passerini and Giugliano during spring, summer, autumn and winter.


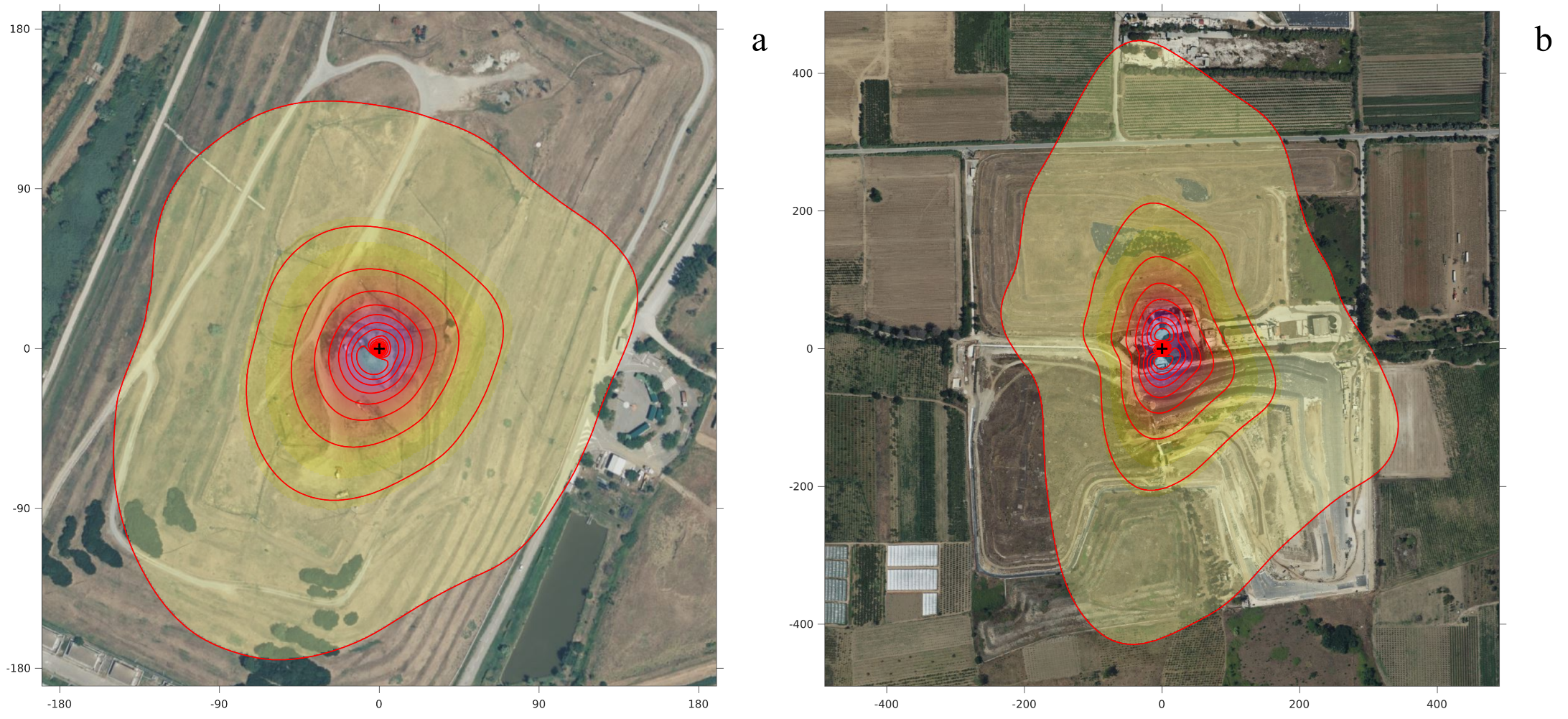


Fig. S1. Map of the flux tower area with footprint raster and contour lines from 10 to 90%, in 10% steps based on the method developed by Kljun et al. (2015) at Case Passerini (a) and Giugliano (b). The analysis revealed that 90% footprint distances are mostly contained within the landfill area.


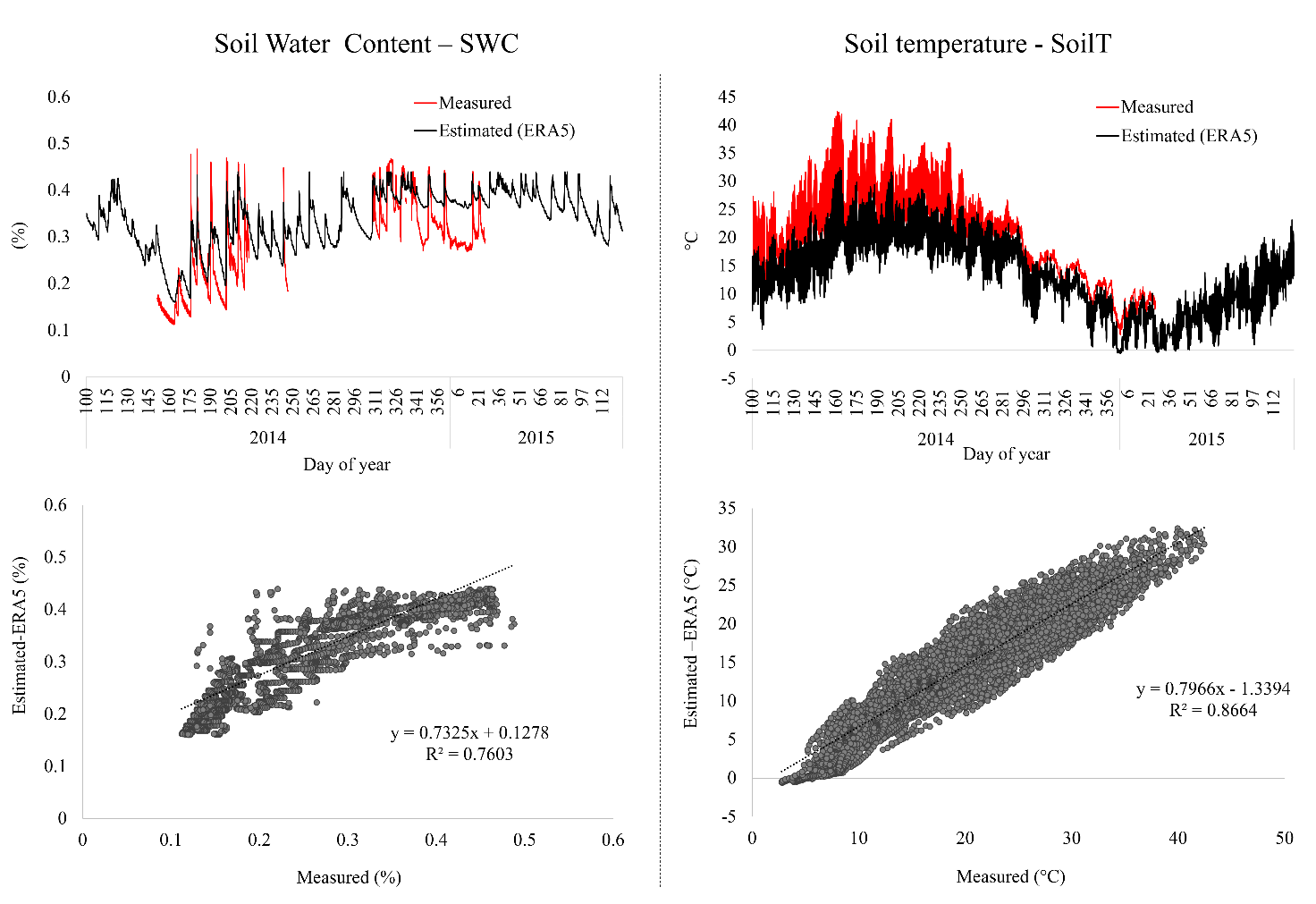


Fig. S2. Hourly pattern and relative scatterplot between soil water content (SWC) and soil temperature data obtained from ERA5-land and SWC and soil temperature measured at Case Passerini.


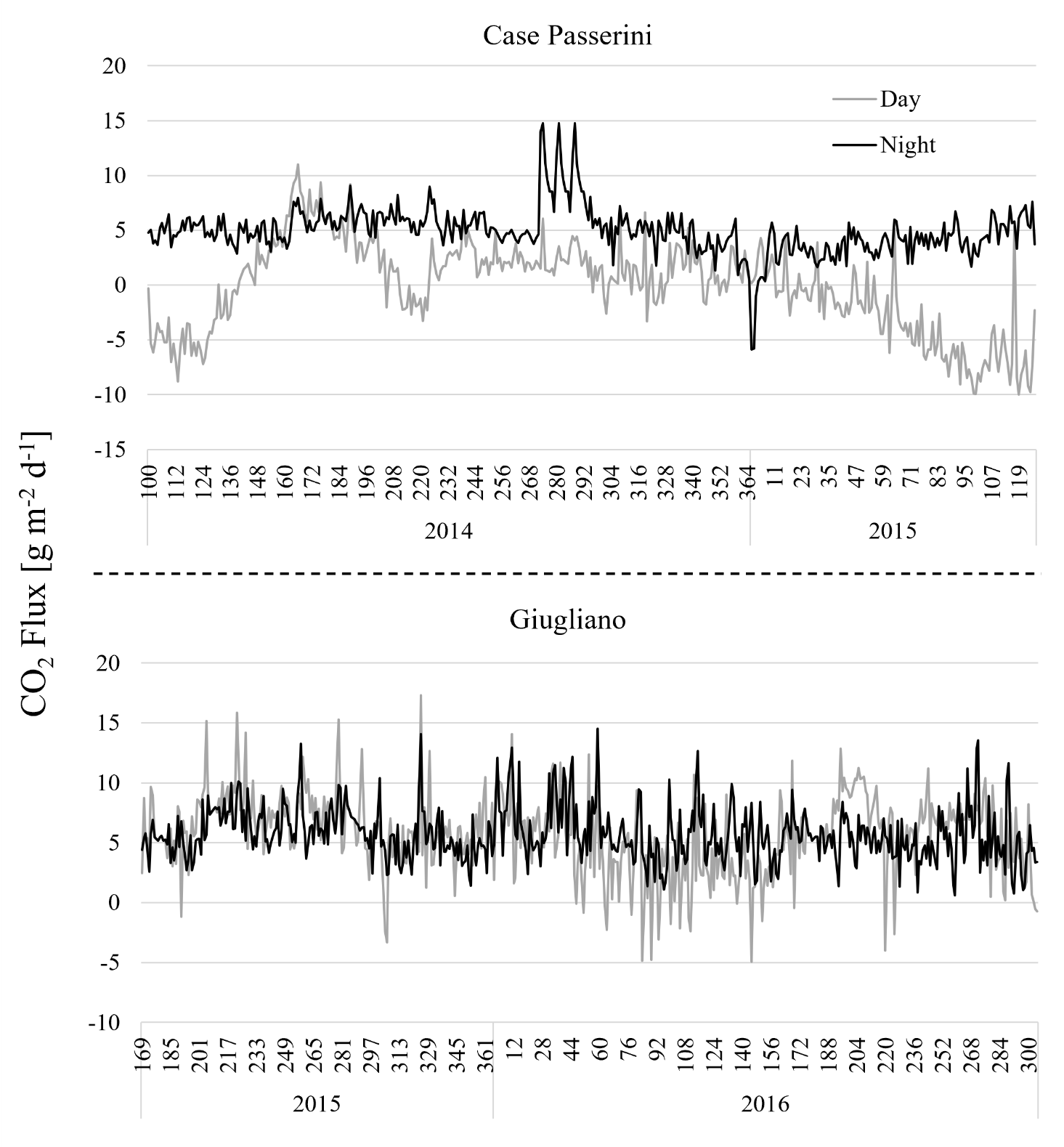


Fig. S3. Nighttime vs daytime daily CO_2_ fluxes at Case Passerini and Giugliano.


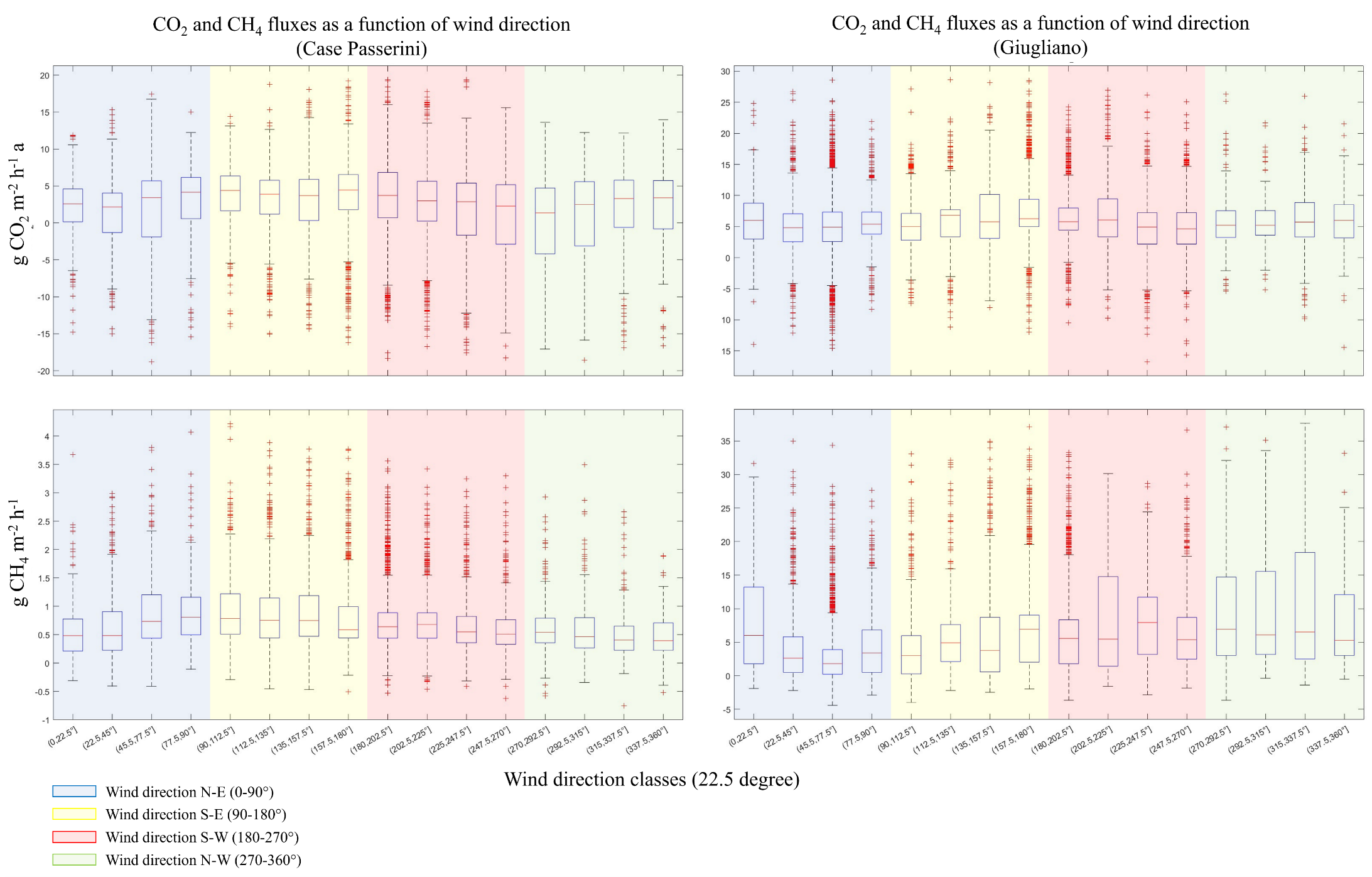


Fig. S4. Boxplot of CO_2_ and CH_4_ fluxes as a function of wind direction grouped at 22.5° and distinguished for wind sectors (i.e., North, Est, South and West) according to the related colour (blue, yellow, red and green). The central line in each box indicates the median, and the bottom and top edges of the box indicate the 25th and 75th percentiles, respectively.


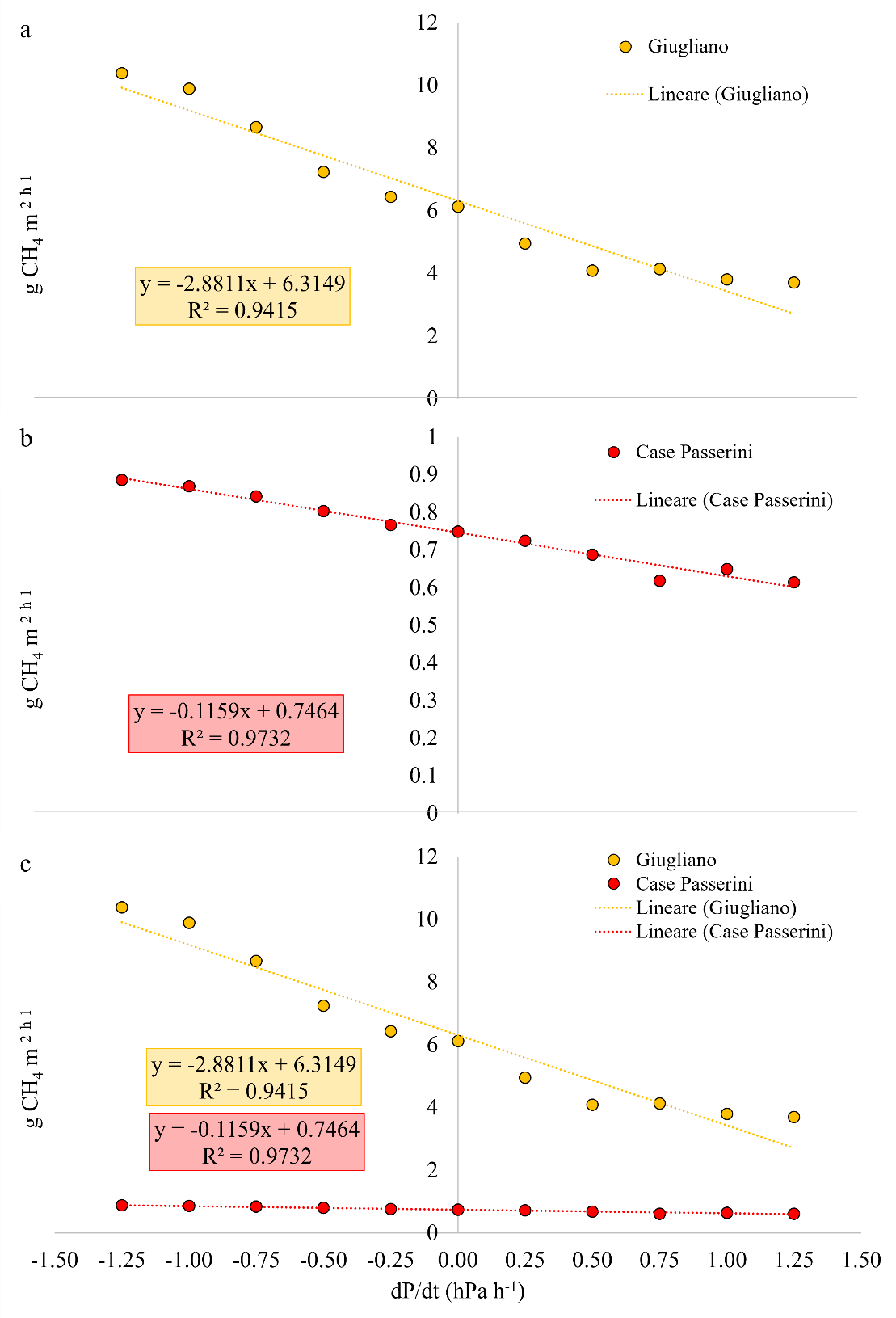


Fig. S5. Relation between average CH_4_ fluxes and dP/dt classes contained a number of samples >1% of the entire dataset at: a) Giugliano; b) Case Passerini, c) Case Passerini and Giugliano.


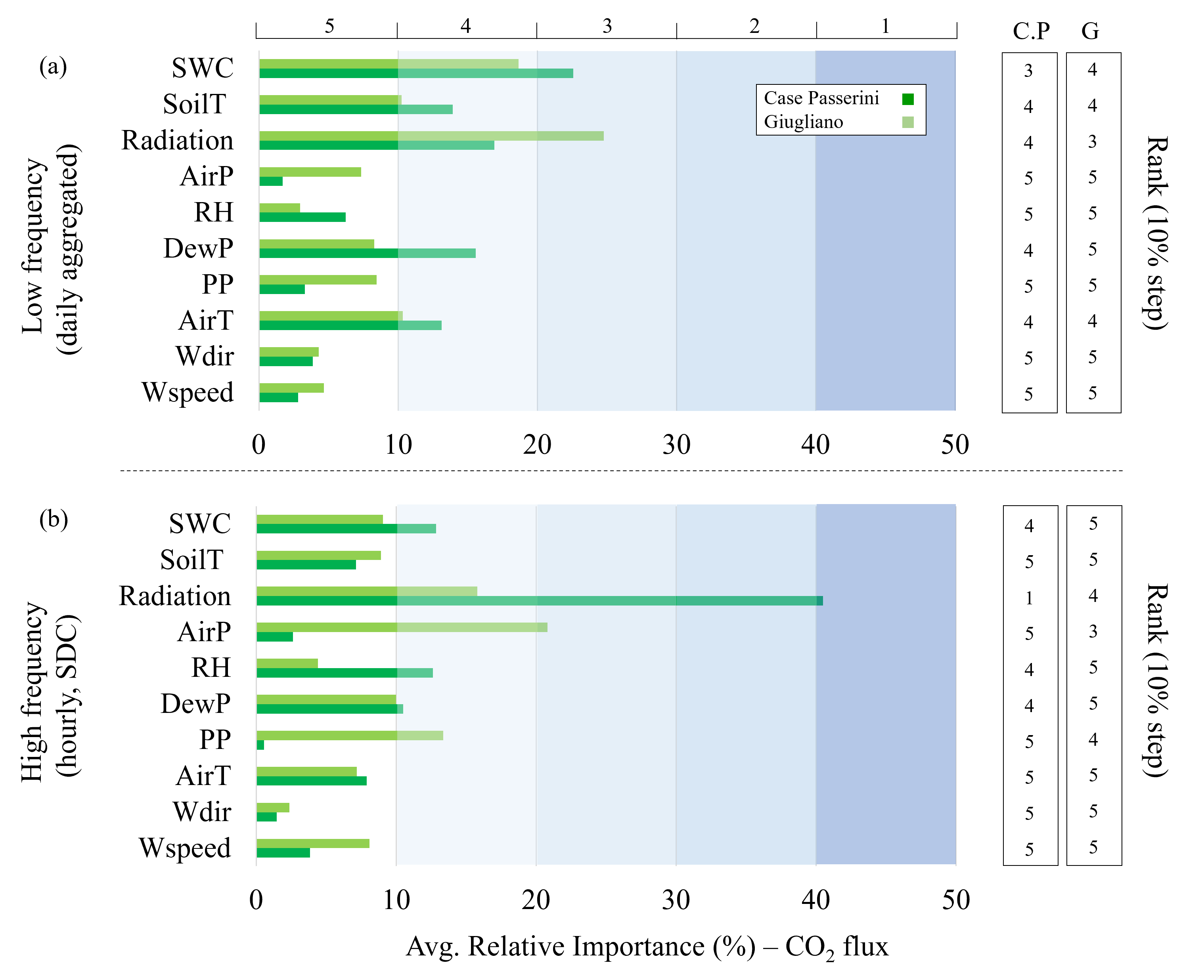


Fig. S6. Relative importance (%) of meteorological variables to CO_2_ fluxes as accounted as average of (a) low-frequency (hourly and SDC) and (b) high-frequency (daily, 5-days, 10-days, 15-days, monthly and seasonal) time-step. The rank of meteorological variables (1 to 7) was empirically defined grouping at 10% step their relative importance.


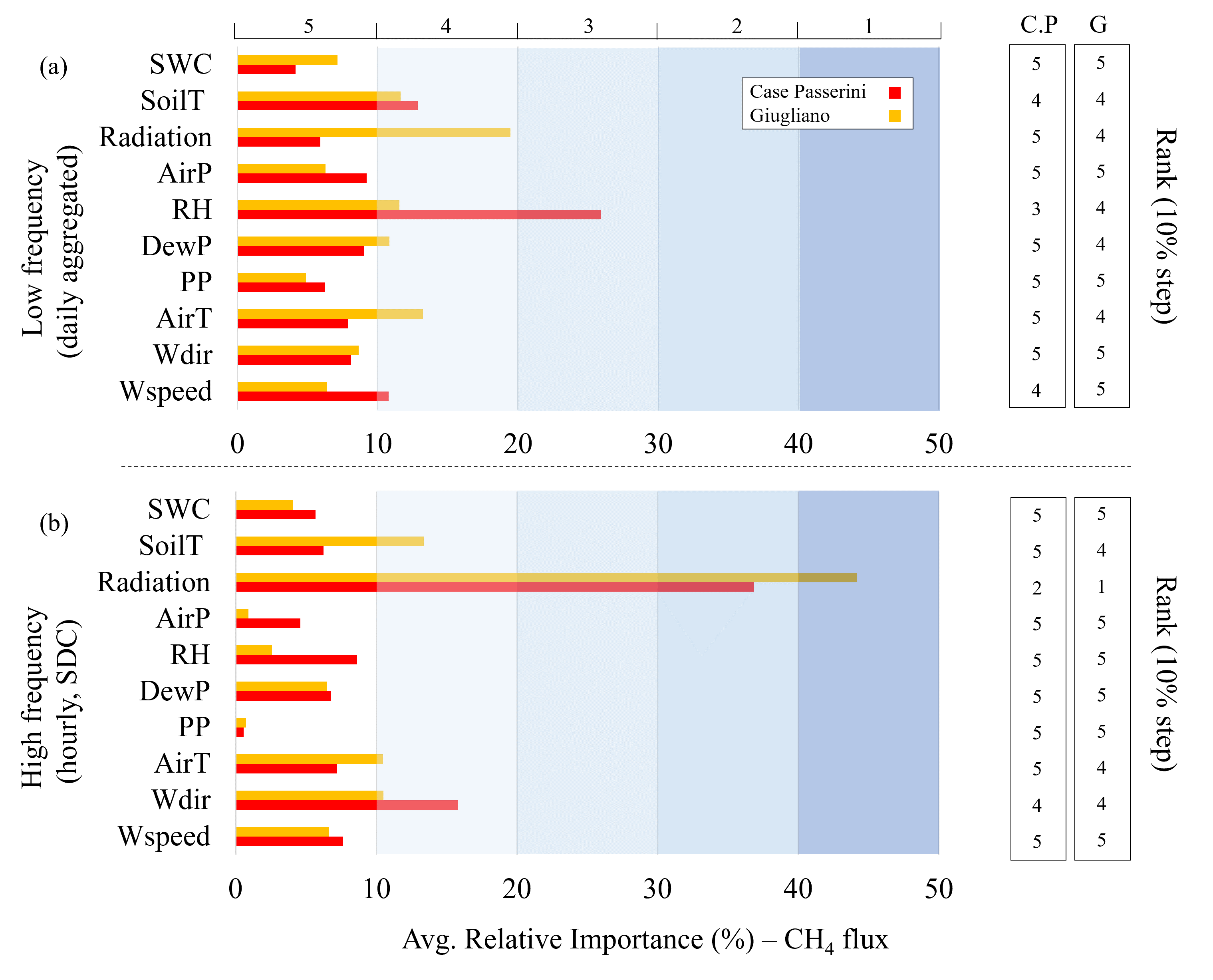


Fig. S7. Relative importance (%) of meteorological variables to CH_4_ fluxes as accounted as average of (a) low-frequency (hourly and SDC) and (b) high-frequency (daily, 5-days, 10-days, 15-days, monthly and seasonal) time-step. The rank of meteorological variables (1 to 7) was empirically defined grouping at 10% step their relative importance.


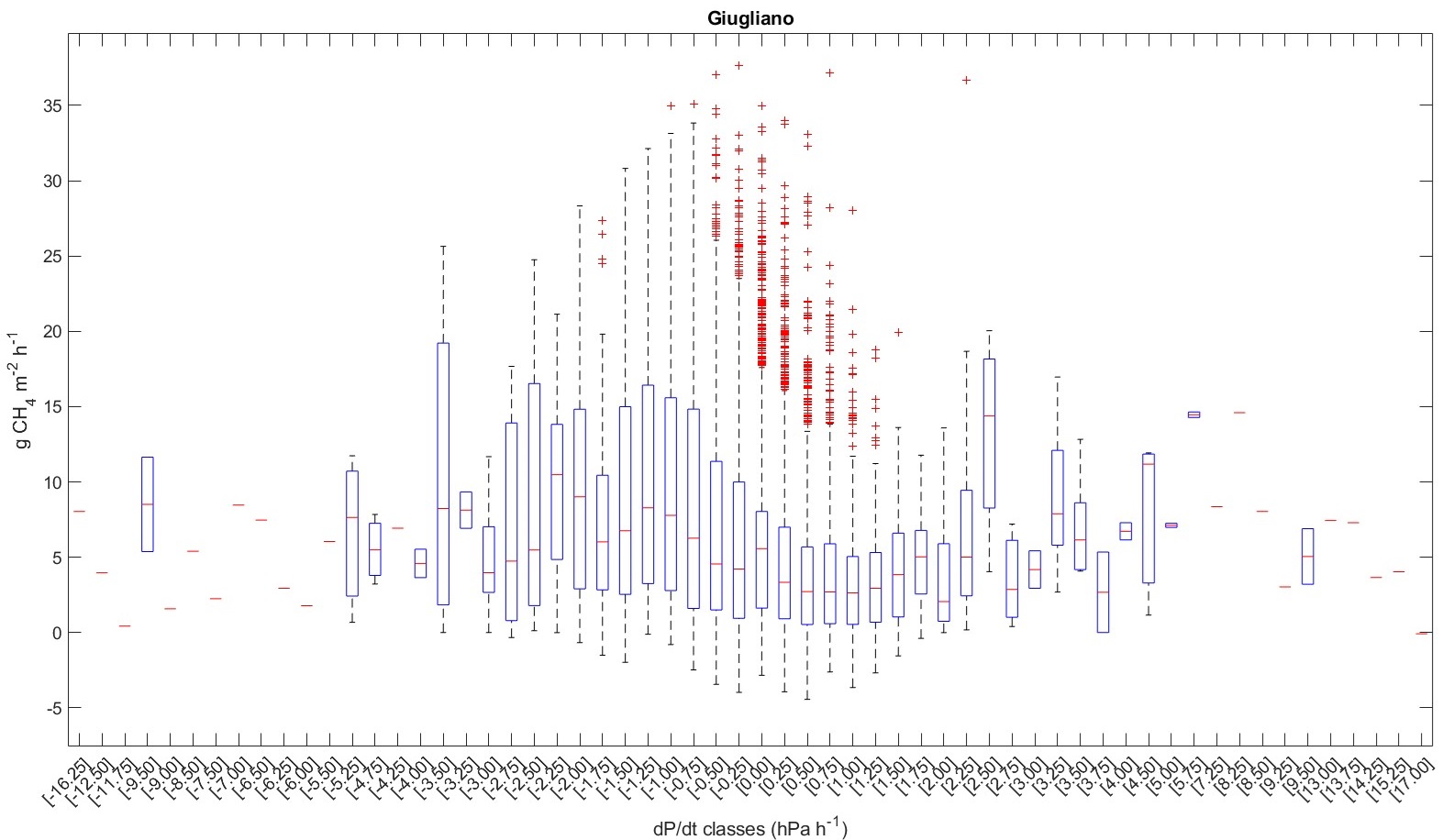


Fig. S8. CH_4_ hourly fluxes grouped in dP/dt classes of 25 Pa at Giugliano. The whole dataset (12029 values) was grouped in 60 dP/dt classes ranging from -16.25 to 17.00.


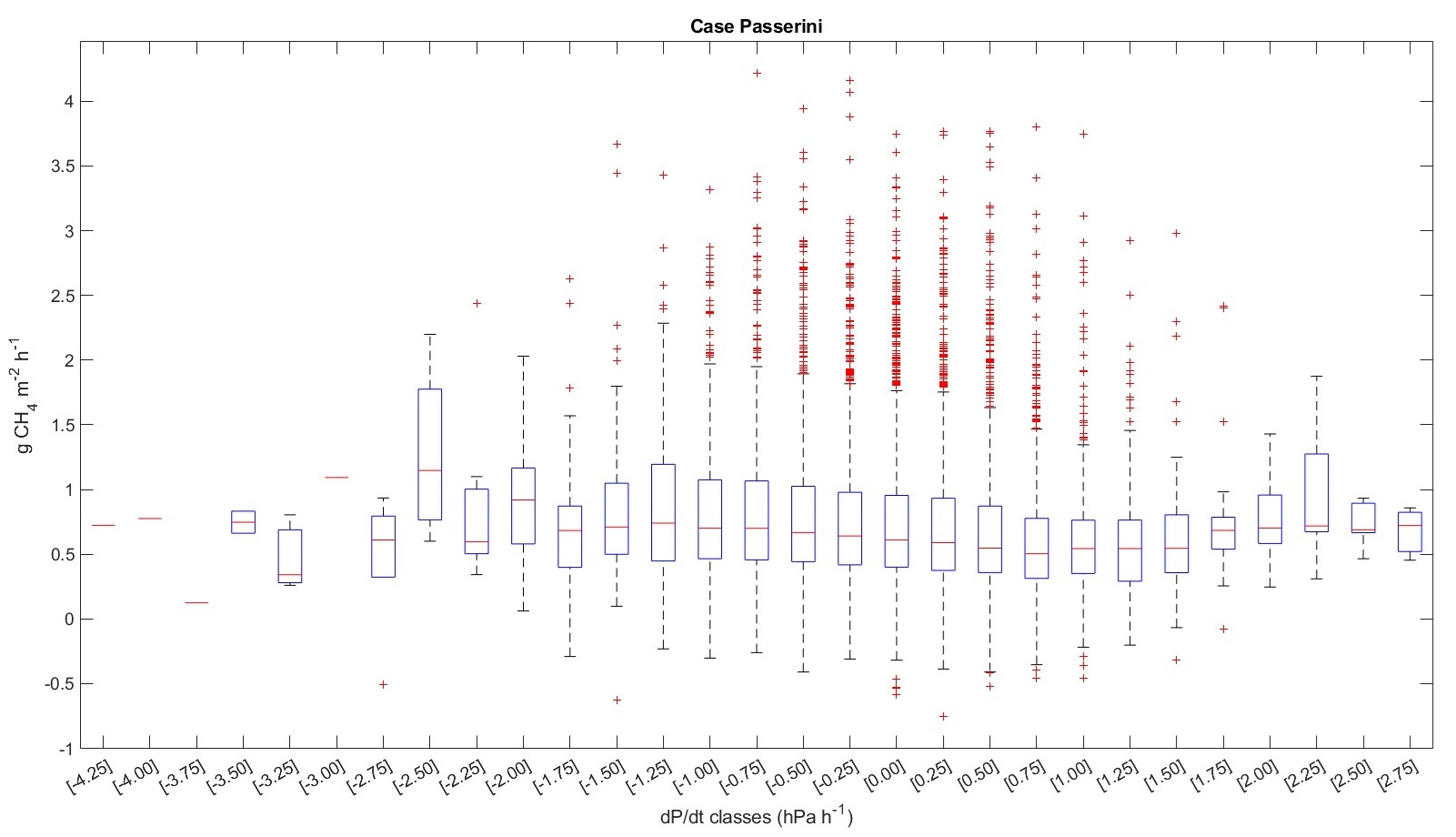
Fig. S9. CH_4_ hourly fluxes grouped in dP/dt classes of 25 Pa at Case Passerini. The whole dataset (9408 values) was grouped in 60 dP/dt classes ranging from -4.25 to 2.75.


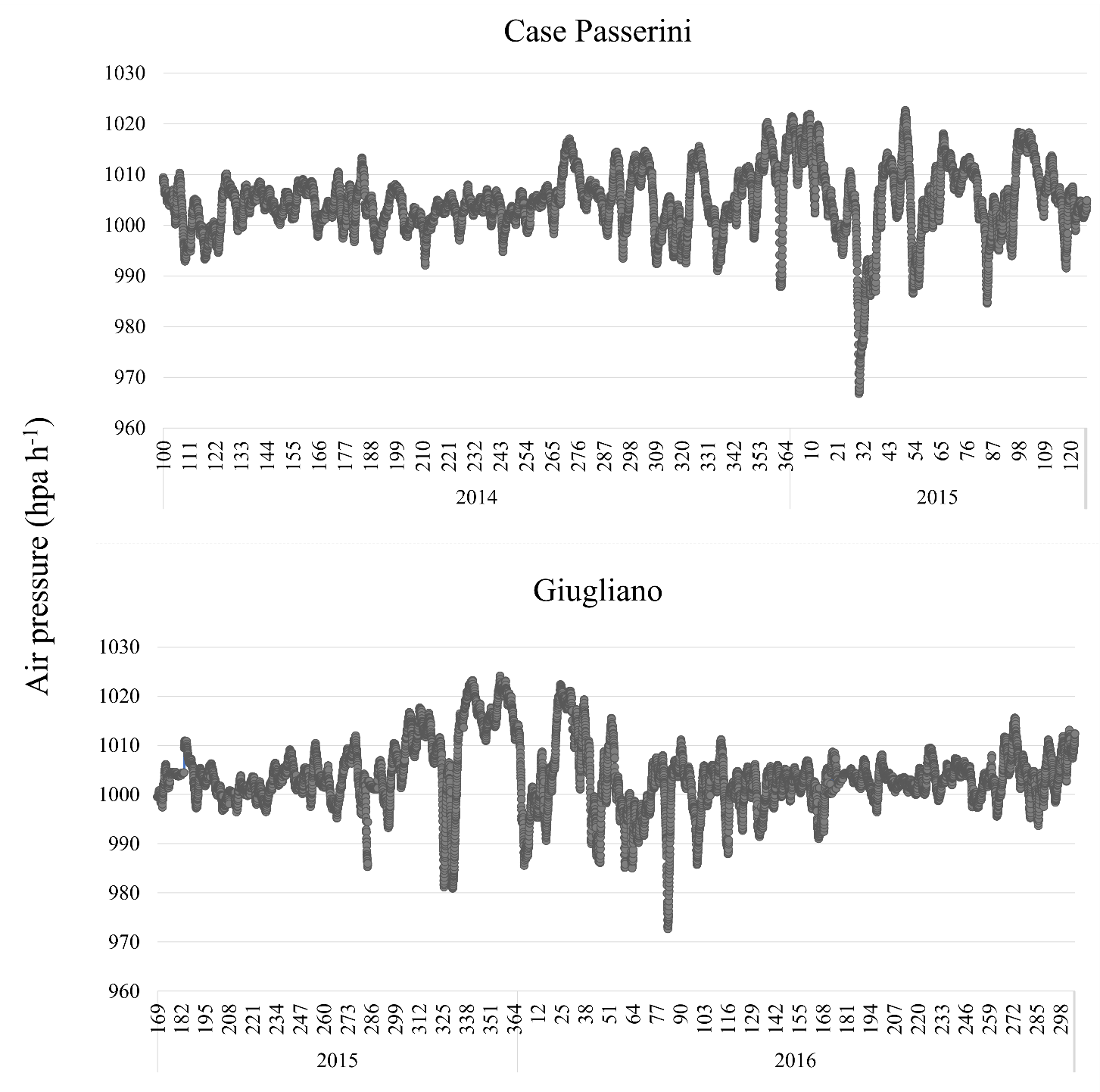


Fig. S10. Hourly air pressure (hPa) at Case Passerini and Giugliano during the study periods.


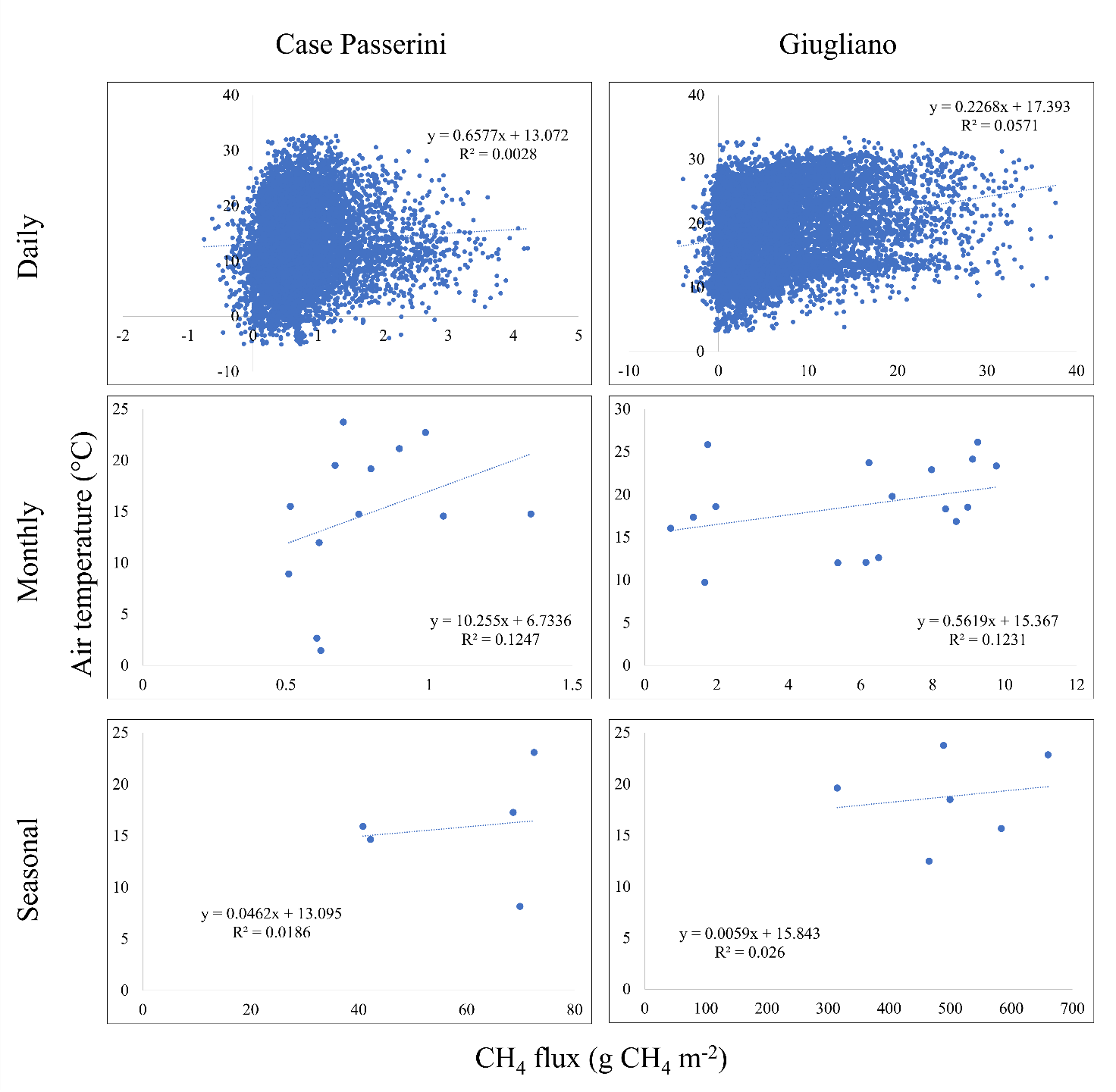


Fig. S11. Correlations between air temperature and CH_4_ fluxes at daily, monthly and seasonal timescale.
